# Low Power FA_2_PbI_4_/SiO_2_ Bilayer Memristors with Pt Nanoparticles Exhibiting Reconfigurable Synaptic and Neuron Properties for Compact Optoelectronic Neuromorphic Systems

**DOI:** 10.1021/acs.nanolett.5c03475

**Published:** 2025-10-06

**Authors:** Panagiotis Bousoulas, Spyros Orfanoudakis, Danai Spathi, Victoras Pagonis, Leonidas Tsetseris, Charalampos Tsioustas, Polychronis Tsipas, Athanassios G. Kontos, Thomas Stergiopoulos, Dimitris Tsoukalas

**Affiliations:** † Department of Physics, School of Applied Mathematical and Physical Sciences, 68994 National Technical University of Athens, Iroon Polytechniou 9 Zographou, 15780 Athens, Greece; ‡ Institute of Nanoscience and Nanotechnology, 54572 NCSR Demokritos, 15341 Athens, Greece

**Keywords:** memristors, neuromorphic properties, Pt nanoparticles, perovskites, light intensity, spikes, synapses, artificial neural networks

## Abstract

The development of artificial neural networks with biorealistic computing properties represents a frontier in the neuromorphic computing era. However, achieving compact and energy-efficient integration of silicon-based synapses and neurons remains challenging due to complexities in their electrical circuits. Herein, we fabricated a low power Ag/SiO_2_/FA_2_PbI_4_/Pt nanoparticles/ITO bilayer memristor with reconfigurable properties, exhibiting dual switching modes and neuromorphic functionalities. These effects were experimentally investigated through transient response and endurance measurements, while valuable insights were provided using a comprehensive numerical model. The SiO_2_/FA_2_PbI_4_ and FA_2_PbI_4_/Pt nanoparticle interfaces played a critical role in regulating ion migration, stabilizing filament dynamics and enhancing device reliability. A compact optoelectronic neuromorphic system was demonstrated by integrating synaptic and neuronal elements, enabling precise control of the firing activity. An ultralow power consumption (∼10 fJ/spike) was achieved, comparable to that of the human brain and state-of-the-art memristive technologies, thereby paving the way for energy-efficient optoelectronic computing platforms.

## Graphical abstract

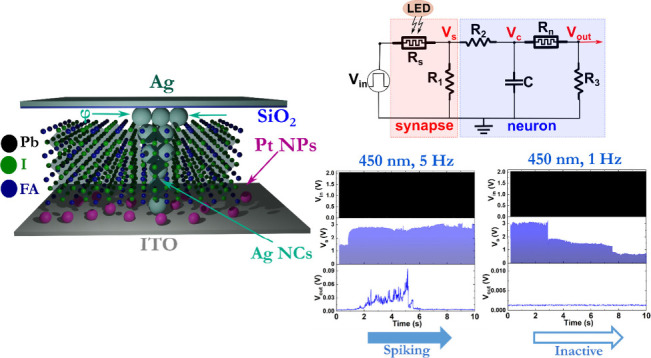

Neuromorphic computing represents a paradigm shift in hardware design, inspired by the architecture and operational principles of biological neural networks. By emulating the brain’s efficiency, parallelism, and adaptability, neuromorphic systems enable energy-efficient computation and real-time learning capabilities for a wide range of applications, including adaptive signal processing, brain-computer interfaces, autonomous robotics, and edge intelligence.^1–4^ However, there are challenges related to the programming complexity and the connection of the various heterogeneous building blocks of artificial neural networks (ANNs). While the former issue can be addressed using specialized tools and design strategies, the latter requires innovation at the device level to bridge the performance mismatch between neuronal and synaptic elements. Reconfigurable neuromorphic systems represent the next leap in efficient and adaptive in-memory computing, as they provide the desired adaptability and flexibility for designing advanced neuromorphic architectures.^5^ Reconfigurability allows on-device adaptation to changing environments (e.g., lighting, noise, sensor drift) and enables constant learning by rewiring synaptic connections dynamically without cloud dependency.

The demonstration of both artificial neural and synaptic activity from the same material configuration is extensively investigated in the literature.^6–19^ However, in most cases, heterogeneous systems comprising synapses and neurons fabricated on different substrates and using dissimilar processes have been reported, which hinders their scalability. In addition, the performance mismatch between the coexisted synaptic and neuronal elements leads to difficulties in the design of the peripheral circuits. The fabrication of dense and efficient ANNs is also closely linked to power consumption, which should be minimized.

To fulfill the above-mentioned stringent requirements, multifunctional devices and materials with intriguing properties are urgently needed. Memristors arise as highly promising candidates for next-generation electronic devices due to their capability to simultaneously store and process information.^20,21^ This unique property permits them to emulate biological synaptic plasticity and neuronal spiking dynamics,^22,23^ making them particularly attractive for neuromorphic computing applications.^24,25^ Recent advances in memristor research have focused on innovative device architectures,^26,27^ enhanced energy efficiency,^28^ and seamless integration with neuromorphic and optoelectronic platforms.^29^

At the same time, optical programming of neuromorphic elements has emerged as a promising strategy for developing light-programmable neuromorphic elements. This approach is expected to play a crucial role in the development of intelligent photonic processors and artificial visual systems.^30,31^ Along these lines, hybrid organic–inorganic, metal-halide perovskites arise as a revolutionary class of materials capable of emulating in-memory photonic computing tasks.^32–35^ They can be fabricated using a range of low-cost and simple techniques, which allow tailoring of the film morphology and crystallinity and provide great perspectives for large-scale production.^36^

In this work, a mixed hybrid two-dimensional (2D)/three-dimensional (3D) metal halide perovskite, namely FA_2_PbI_4_ (where FA stands for formamidinium cation), combined with a thin SiO_2_ layer (10 nm) on top was adopted, resulting in a stable operation of the memory device for more than 4 months, which has not been reported elsewhere for FAPI-based memristors. The influence of the SiO_2_ capping film thickness and the Pt nanoparticles (NPs) layer on the device performance was systematically examined. Numerical simulations were also deployed to investigate the formation origins of the percolating conducting filament (CF). A transition from volatile to nonvolatile pattern was captured by increasing compliance current (I_cc_), which was leveraged to emulate the artificial neuron and synaptic properties. The latter properties were totally attained under light irradiation through the transparent bottom electrode (BE), yielding a power consumption per synaptic weight of ∼20 nJ. An even lower power consumption of ∼10 fJ/spike was extracted during the emulation of the artificial neuron properties using a simple RC circuit. These robust neuromorphic units were seamlessly integrated to form a homogeneous computing system, exhibiting cognitive capabilities enabled by optical stimulation at three different wavelengths. In particular, photonic synapses were dynamically adjusted using light pulses and the excitation of leaky integrate-and-fire (LIF) neurons with adaptive thresholds was accurately controlled. This mimics biological neurons’ refractory periods, ensuring continuous firing and subsequent reset operation without using inhibitory feedback circuits. Together, the interplay between adaptive synapses and responsive artificial neurons allows the system to perform real-time signal processing, learning, and decision-making tasks in a manner analogous to the human brain.

The structural, optical, and chemical characterization of the perovskite film provides clear evidence for the formation of a mixed-phase material comprising both 2D FA_2_PbI_4_ and 3D α-FAPbI_3_ domains.^37^ The X-ray diffraction (XRD) patterns (Figure 1a) exhibit the characteristic features of both 2D and 3D perovskite structures. Low-angle reflections were observed at approximately 6.5°, 9°, and 11°, which are assigned to the layered structure of FA_2_PbI_4_. The reflection at ∼6.5° corresponds to the (00l) planes of the n = 1 Ruddlesden–Popper (RP) phase, with an interlayer spacing of ∼13–14 Å, while higher-order reflections at ∼9° and ∼11° further confirm the presence of well-ordered 2D layered domains.^38^ In addition, intense reflections at ∼14°, ∼23.5°, and ∼28° are characteristic of the (001), (012), and (002) reflections of the 3D α-FAPbI_3_ phase, indicating a significant contribution from 3D crystallites in the film.^39^

**Figure 1:**
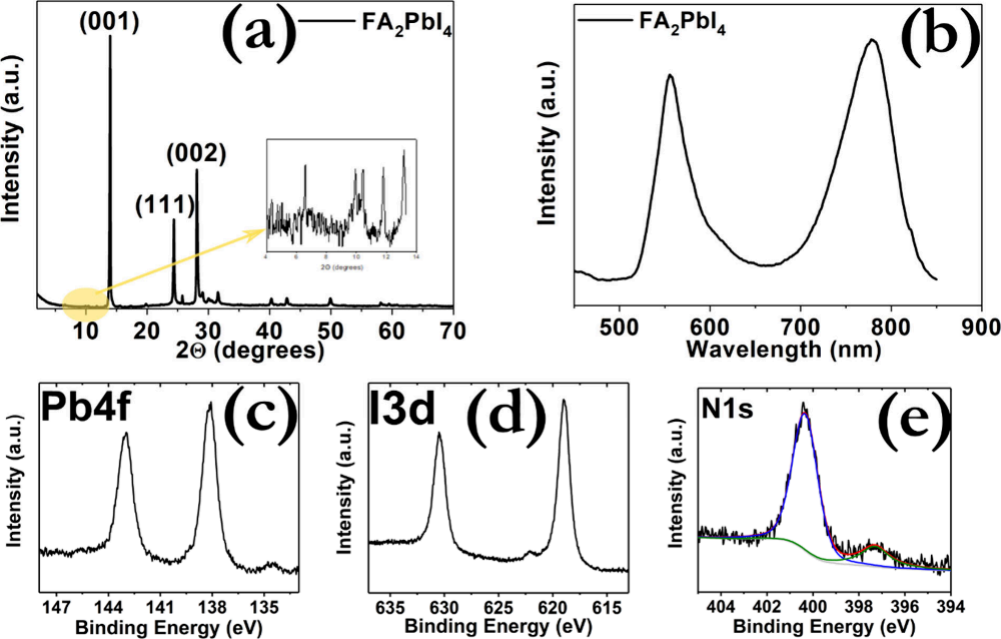
(a) XRD diffractogram of the ITO/FA_2_PbI_4_ film. (b) Photoluminescence spectrum of the ITO/FA_2_PbI_4_ film. (c) Pb 4f, (d) I 3d, and (e) N 1S spectra with the corresponding peaks after fitting analysis.

The coexistence of low- and high-angle reflections clearly demonstrates a mixed-phase structure. Photoluminescence (PL) spectroscopy (Figure 1b) further corroborates this interpretation. The PL spectrum exhibits two dominant emission peaks at ∼570 nm and ∼790 nm. The ∼570 nm emission is assigned to excitonic recombination from FA_2_PbI_4_, typical of strongly bound excitons in the 2D layered structure.^40^ The ∼790 nm emission corresponds to band-edge recombination in α-FAPbI_3_.^39^ The UV–vis absorption spectroscopy (Figure S1) provides additional evidence for the presence of both 2D and 3D perovskite phases in the film.^41^ The simultaneous observation of a well-defined excitonic peak and extended band-edge absorption implies the formation of a mixed-phase system containing both 2D and 3D perovskite domains.^42^ X-ray photoelectron spectroscopy (XPS) (Figure 1c-e and Figure S2) offers a comprehensive chemical analysis of the perovskite film. The I 3d spectrum shows two well-defined peaks at 630 eV (I 3d_3/2_) and 618 eV (I 3d_5/2_), consistent with iodide bound to Pb^2+^ in perovskite structures.^43^ The Pb 4f spectrum exhibits two peaks at 138 eV (Pb 4f_7/2_) and 143 eV (Pb 4f_5/2_), characteristic of Pb^2+^ in a perovskite environment, with no evidence of metallic Pb (which would appear at a lower binding energy).

The N 1s spectrum reveals a prominent peak at 400.5 eV, corresponding to nitrogen in the FA^+^ cation, and a smaller peak at 397.5 eV, which may be attributed to minor decomposition products such as NH_3_ or nitrogen bound in defects. The formation of a mixed-phase system can be rationalized by considering the crystallization kinetics. Although the solution stoichiometry favors the formation of 2D FA_2_PbI_4_, kinetic factors such as solvent evaporation rate, ion diffusion, and limited annealing time can promote concurrent nucleation of 3D α-FAPbI_3_.^44^ The 2D phase can be promoted by using an excess amount of FAI (e.g., 2:1 FAI:PbI_2_ ratio) or applying lower annealing temperatures to suppress the formation of 3D domains. A strong n-type doping was also extracted from the ultraviolet photoelectron spectroscopy (UPS) measurements (Figures S3,S4).

A schematic cross-section of the fabricated devices is presented in Figure 2a,b,d. The devices operated under both switching modes, namely volatile and nonvolatile, by increasing the I_cc_, while a forming step was not necessary prior to device operation (Figure 2c,f). A memory window bigger than 10^6^ was recorded for the biggest applied I_cc_ of 100 μA, while the switching slopes are also quite steep (<50 mV/dec­(A)). The coexistence of dual switching modes accompanied by such large switching ratios has not been previously reported for FAPI-based memristors (Table S3).^45–51^ A robust direct current (DC) endurance behavior for both switching patterns (Figure 2g,h) and alternating current (AC) behavior for the nonvolatile mode (Figure 2i) was detected, indicating the beneficial role of the thin SiO_2_ layer. Indeed, the reference samples that did not contain the Pt NPs and the SiO_2_ protective layer showed a poor endurance behavior and unstable switching patterns (Figure S5). Although the presence of the Pt NPs yielded steeper switching slopes,^52–55^ the endurance pattern remained unstable (Figure S6). When a SiO_2_ layer was added above the FA_2_PbI_4_ film, the endurance patterns became noticeably more stable with respect to the two previous cases (Figure S7). A transmission electron microscopy (TEM) image of the deposited nanoparticles is presented in Figure S8.

**Figure 2:**
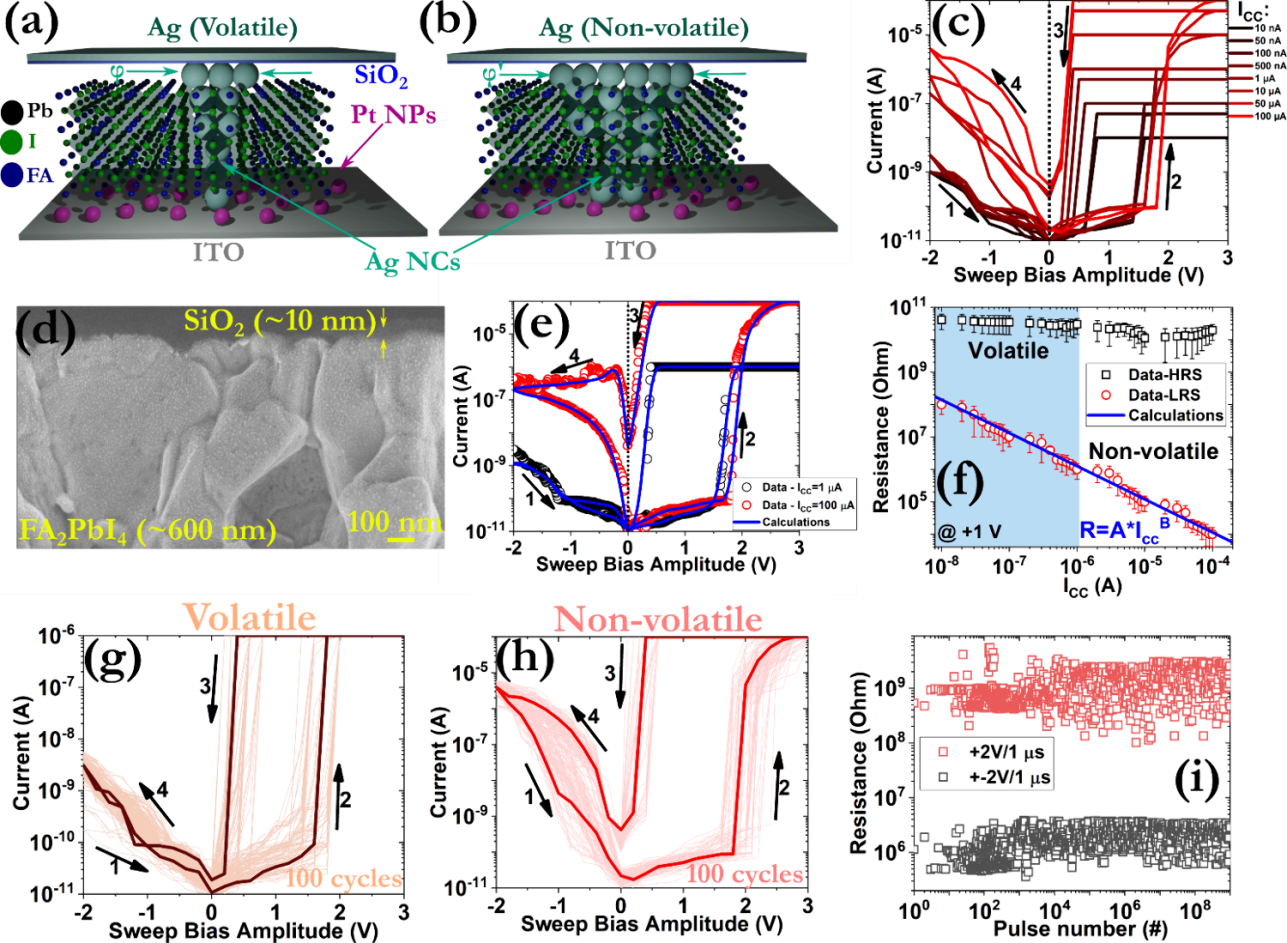
Schematic illustration of the proposed device configuration during the manifestation of the (a) volatile and (b) nonvolatile modes. The symbol φ represents the effective diameter of the CF. (c) I–V hysteresis patterns under the application of various I_cc_ with a voltage scan rate of 200 mV/s. The numbers and arrows in the graph signify the switching direction. Similar hysteresis patterns were obtained by starting the voltage scans from 0 V to either positive or negative biases. (d) Cross-sectional scanning electron microscopy (SEM) image of the FA_2_PbI_4_/SiO_2_ interface, revealing the existence of the 3D domain in the perovskite structure. The 3D domains provide efficient carrier percolation pathways, minimize interlayer hopping resistance, and reduce trap-assisted recombination, improving thus the overall conductivity and switching dynamics. The light absorption and exciton dynamics are also affected by the 3D domains, locally lowering the exciton binding energy and enabling more effective exciton dissociation. (e) Measured and calculated current responses under the application of two I_cc_ values. (f) Measured and calculated distribution of the low resistance state (LRS) as a function of I_cc_. I–V consecutive cycling behavior after the implementation of 100 DC endurance cycles for the (g) volatile and (h) nonvolatile modes. (i) Pulse endurance measurements under the application of alternative pairs of +2 V/1 μs and −2 V/1 μs square pulses. The read-out process was carried out using square pulses of 200 mV/1 μs.

Valuable insights regarding the origins of the switching mechanism are presented in Figure S9, where the independence of the LRS from the total device area indicates the existence of percolating CFs, while the temperature pattern suggests their metallic nature. The devices remained fully operational after a period of 4 months, exhibiting small device-to-device variations (Figure S10). In addition, considering that the dependence of the LRS on the various applied I_cc_ yielded a slope close to 1 (Figure 2f), a previously reported self-consistent numerical model was used to reproduce the switching patterns (Figure 2e).^56^ The basic assumption of this model is that the migration of silver ions, which is dictated by the drift, diffusion, and thermo-diffusion fluxes, leads to the formation of extended chains of metal nanoclusters (NCs) due to the increased solid-solubility of silver and the subsequent material precipitation.^57^ There are also recent experimental proofs concerning the formation of CFs in mixed organic–inorganic halide perovskites.^58^

The simulated geometry and the respective differential equations are presented in Figures S11 and S12, respectively, while the various simulated characteristics during the SET and RESET transitions are provided in Figures S13 and S14. The proposed model can also emulate the dual switching modes, as well as transient responses (Figure S15), taking into account the impact of the BE on the dissipation of the generated heat.^59^ In particular, the small thermal conductivity values of ITO and Pt NPs are not conducive to efficiently removing the generated heat from the device’s active core, which could be as high as 780 K (Figure S16). Considering that the melting point of Ag NCs presents a strong dependence on their diameter,^60^ it can be inferred that the self-rupture of the CF due to the local Joule heating could explain the volatile mode of our devices. This assumption is also compatible with the experimentally observed extremely short relaxation times (Figure S17). In this case, a gap region between the tip of the thin CF and the BE is induced, leading to the spontaneous switch of the device to the high resistance state (HRS) without altering the polarity. On the contrary, a thicker CF, in terms of diameter (φ́>φ - induced by the application of a larger I_cc_), is less susceptible to the local temperature distributions described above and cannot be easily ruptured. The presence of Pt NPs creates localized regions of high surface energy with strong Ag–Pt binding affinity and induces a local electric field enhancement. This reduces the local activation energy (E_drift_) for Ag ion migration near the NPs, making them preferential nucleation sites for Ag accumulation.

The model was also modified to examine the influence of perovskite degradation on the memory performance. Intrinsic material factors were considered, such as ion migration-induced phase segregation using the Cahn–Hilliard formalism.^61–63^ The results revealed negligible degradation at normal values of the coupling coefficient for phase segregation-induced shrinkage of the CF, which is in direct agreement with our measurements across various time periods (Figure S10b), while at extreme cases, a transition from bipolar to threshold mode is predicted (Figure S18). However, the latter effect was only experimentally observed for small I_cc_.

The dual switching modes can be leveraged to emulate various neuromorphic functionalities of the respective biological systems.^64^ To this end, biological synapses allow neurons to transmit signals to one another (Figure 3a). Here, the excitatory postsynaptic current (EPSC) responses were recorded after being irradiated with different light colors (blue, red, and green) from the bottom transparent electrode (Figure 3b). As shown by the normalized data in Figure 3c, the device’s output can be continuously tuned (i.e., synaptic potentiation) using high frequency optical pulses (5 Hz), whereas lower frequencies induced synaptic depression. The source data can be found in Figure S19, while measurements under dark conditions were conducted, confirming that the observed modulation arises from the photo induced carried dynamics (Figure S20).

**Figure 3:**
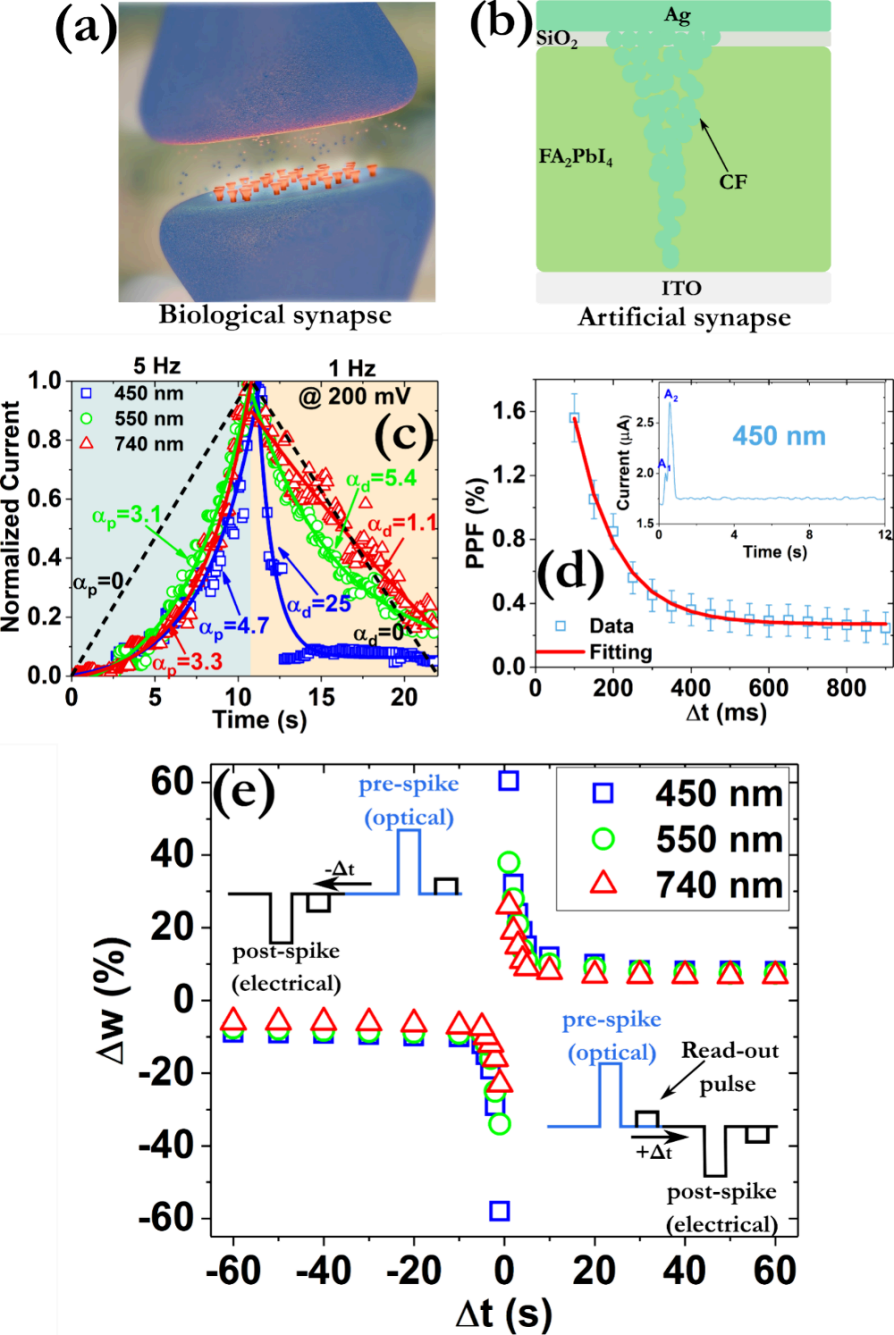
(a) Image of a biological synapse. (b) Schematic cross-section of the device configuration. (c) Normalized data indicating the continuous modulation of the conductance states during consecutive device operation under light irradiation with various wavelengths and different frequencies at a constant light intensity of 1.4 mW/cm^2^. The photocurrent at ∼740 nm can be attributed to minority lower-bandgap domains—either 3D FAPbI_3_-like inclusions or higher-n quasi-2D phases—that are known to form in mixed-dimensional films and to dominate the long-wavelength response. (d) Distribution of the paired pulse facilitation (PPF) index as a function of the pulse interval (Δt) of the optical pulses under constant irradiation with an intensity of 1.4 mW/cm^2^ and duration of 100 ms. The red solid line denotes the fitting by an exponential function. The inset indicates the recorded EPSCs profiles with an optical pulse interval of 100 ms. The parameters A_1_ and A_2_ represent the intensities of the EPSC peaks imposed by the first and second optical pulse, respectively. (e) STDP patterns using various wavelengths. All EPSCs responses were recorded at a read-out voltage of 200 mV.

Smoother transitions were captured during the irradiation with the green (550 nm) and red (740 nm) light sources, in contrast to the more abrupt synaptic characteristics induced by the blue light (450 nm). The high density of photogenerated electrons in the latter case induces a downward band bending in the FA_2_PbI_4_ conduction band near the SiO_2_/FA_2_PbI_4_ interface, effectively reducing the barrier height for electron injection into the SiO_2_ layer at positive biasing (Figure S21). The fully optical control of the synaptic weight update process provides unique opportunities to reduce the complexity of peripheral circuits, as no electrical stimuli are required.^65–68^ Color-selective learning was demonstrated by implementing spike-timing-dependent plasticity (STDP)-based training on RGB-pattern recognition tasks using multicolor optical pulses (Figure 3e and S22).

The electrical power consumption of the produced EPSCs was also estimated using the following equation:

1 $$E_{electrical}^{EPSC}=V\times I\times t_{spike}$$

where V is the amplitude of the voltage pulse (200 mV), I is the current change of the EPSCs (∼1 μA) and t_spike_ is their width (∼100 ms). A power consumption of ∼20 nJ was extracted for the blue optical pulse (450 nm), while even smaller power consumptions were calculated for the other two light sources. These experimentally reported values are among the lowest that have been reported in the literature for FAPI-based perovskite memristors.^69^ A pair of presynaptic optical pulses was applied and the influence of their time interval (Δt) on the PPF index (A_2_/A_1_) was calculated (Figure 3d and Figure S23).^70,71^

For emulating the properties of biological neurons (Figure 4a), devices with volatile characteristics are required (Figure 4b).^72^ Square-shaped electrical pulses with a constant width of 980 μs and a delay of 20 μs were delivered at the input of the artificial neuron (Figure 4d). When the V_c_ exceeds the threshold voltage of the memristor, the capacitor discharges and a voltage spike is recorded at the output resistor due to the switch of the memristor to the ON-state (Figure 4c). The discharge of the capacitor can take place in multiple steps considering that the refractory period (i.e., the time required to fully relax the generated spike to the initial state – 30 ms) is bigger than the RC time constant of the charging loop (τ_RC_ = 10 ms). The spikes are randomly generated and an increase in the applied electrical stimulus leads to an elevated number (Figure 4e). A sigmoid activation function can be used to study the number of generated pulses, indicating their stochastic nature (Figure 4f):

2 $$N_{spikes}=D_{2}+\frac{\left(D_{1}-D_{2}\right)}{\left(1+e^{(V_{in}-V_{0})/V}\right)}$$

where *D*_1_, *D*_2_, *V*_0_, and *V* are fitting parameters and *V*_*in*_ is the input voltage. A low power consumption of ∼10 fJ per spike was estimated. Initially, when the memristor is in the OFF state, the current *I*_*IN*_ is about equal to the current *I*_*C*_ that passes through the capacitor C considering the very high resistance of the memristive loop:

3 $$I_{IN}\approx I_{C}=C\frac{dV}{dt}=\frac{CV_{IN}}{\tau_{RC}}e^{-t/\tau_{RC}}$$

where *t* is the time needed for the memristor to turn ON (∼0.3 s). When the memristor switches to the ON state, the current that will pass through, will be about equal to the current (*I* ∼ *I*_*IN*_) and the energy consumption per spike can be estimated through eq 1 (*t*_spike_∼50 ms).

**Figure 4:**
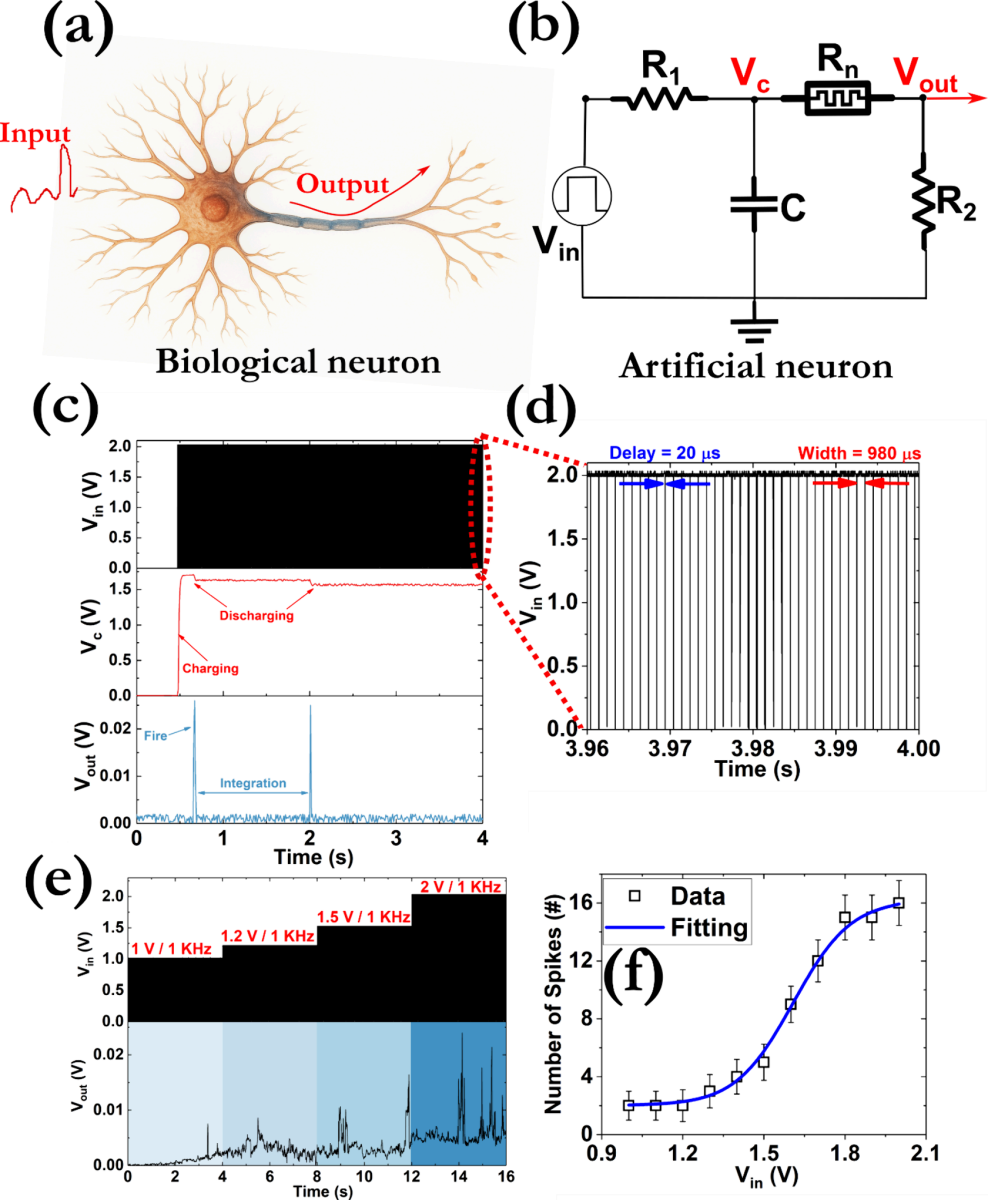
Schematic illustration of (a) a biological and (b) an artificial neuron to obtain the LIF response. For the latter, a simple RC was used, consisting of an input resistor (R_1_ = 100 kOhm), a charging capacitor (C = 100 nF), the volatile memristor (R_n_), and the output resistor (R_1_ = 100 kOhm). (c) Experimental results of the evolution with the time of the input voltage (V_in_ - black line), the voltage drop across the capacitor (V_c_ – red line), and the voltage at the output of the circuit (V_out_ – blue line). (d) Distribution of the electrical input square pulses depicting their width (980 μs) and delay (20 μs). The applied frequency was 1 kHz. (e) Impact of the input voltage amplitude on the LIF neuron activity for a period of 16 s. (f) Distribution of the generated spikes as a function of the amplitude of the input voltage. The data have been collected by measuring 100 different artificial neurons.

The large number of neurons—estimated at around 86 billion—and their intricate connectivity allow for a high degree of parallel processing and integration of sensory information.^73^ A simplified diagram highlighting the neural pathways is provided in Figure 5a. The preneuron emits a train of spikes that induce an increase in the synapse’s action potential. Once the latter exceeds a threshold value, the postneuron is activated by generating its own spikes, achieving thus the successful transmission of the signal from the preneuron. The training of the synapses can be accelerated by using optical pulses, yielding a faster and more precise control of the neural activity with a lower power consumption. A schematic illustration of the used optoelectronic neuromorphic system is displayed in Figure 5b and the applied protocol can be seen in Figure 5c. Figure 5d presents the voltage distributions across the artificial synapse (V_s_) and neuron (V_out_) modules. When only an input electrical pulse with an amplitude of 2 V and frequency 1 kHz was delivered, no spikes at the output neuron were recorded (Figure S24). When the synapses (R_s_) are irradiated, their conductance increases at a greater rate and the same pattern follows the V_s_, according to the voltage division principle. The irradiation with red light at 740 nm yielded the spike generation from the neuron circuit at about 4 s and with an average amplitude of 25 mV. The amplitude of the generated spikes was increased to about 40 and 90 mV when the devices were irradiated with green and blue light sources, respectively, while the time-to-first-spike was also reduced (Figures 5e,f). For the deactivation of the neurons, the frequency of the optical signal was reduced from 5 to 1 Hz (Figure S25), while the polarity of the input electrical signal was not changed. These processes were repeated several times, providing an efficient route for the optical excitation and inhibition of synapses and the subsequent control of the firing activity. The enhanced performance of our prototype can be ascertained from the benchmark analysis including several device and system level metrics (Table S4).

**Figure 5:**
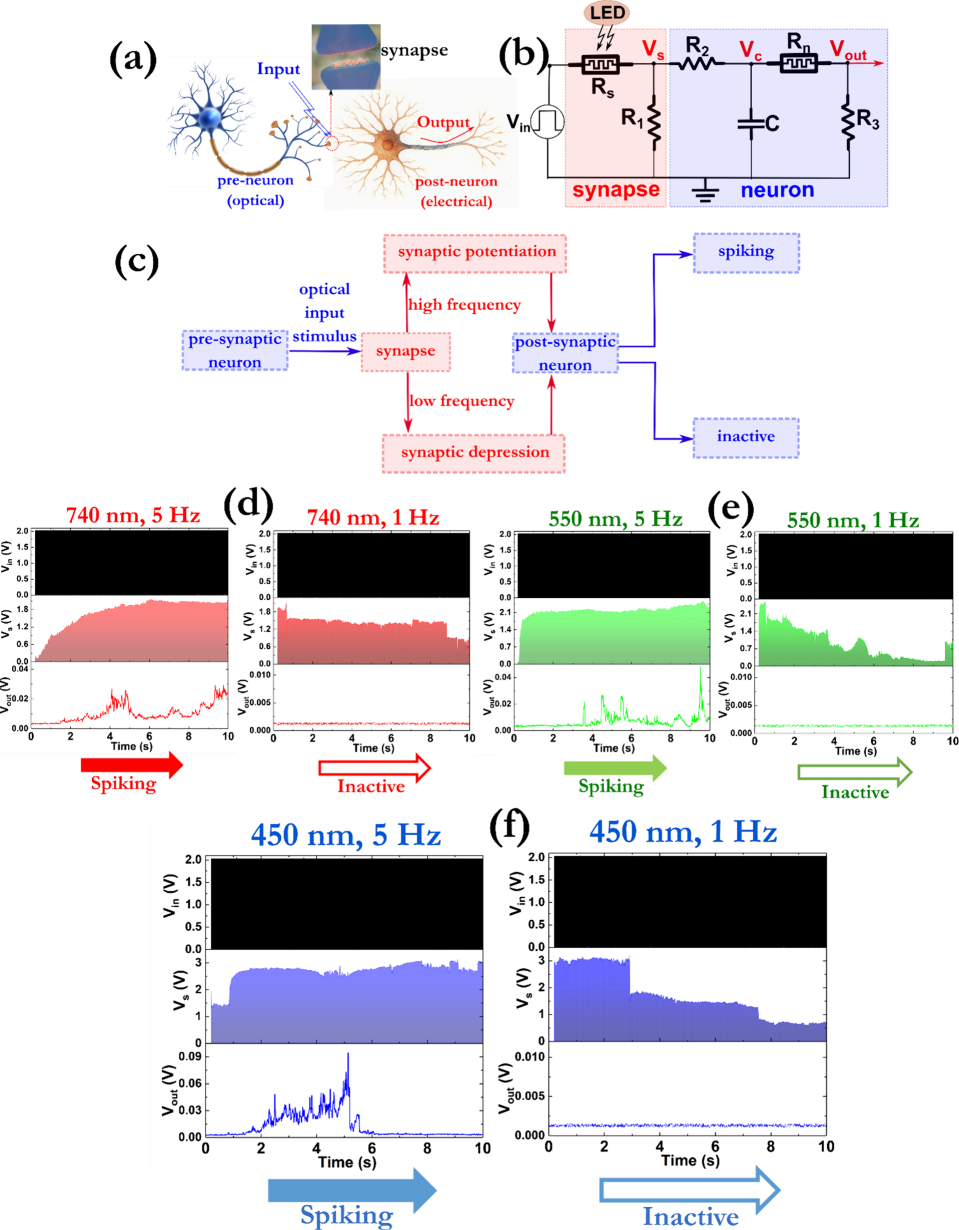
Depiction of a compact and homogeneous optoelectronic neuromorphic system consisting of (a) biological neurons and synapses and (b) artificial neurons and synapses (R_1_ = 40 kOhm, R_2_ = R_3_ = 100 kOhm, C = 100 nF). (c) Diagram of the process used to program the various devices. (d)–(f) Experimental results of the evolution with time of the input voltage (V_in_ - black line), the voltage drop across the synapse (V_s_ – red, green, blue lines), and the voltage at the output of the circuit (V_out_ – red, green, blue lines). The amplitude and the frequency of the input voltage were always 2 V and 1 kHz, respectively, while optical pulses with different frequencies (5 and 1 Hz) and the same width of 100 ms were applied to induce the synaptic potentiation and depression effects.

In this work, a robust Ag/SiO_2_/FA_2_PbI_4_/Pt NPs/ITO memristor with optoelectronic neuromorphic properties and dual switching modes was presented. The volatile nature was systematically investigated to emulate the properties of biological neurons, while the nonvolatile behavior was leveraged to reproduce artificial synaptic properties using only light stimuli. The property of accurately tuning the synaptic weight of our devices was further exploited to build a homogeneous neuromorphic computing system comprising both neuronal and synaptic elements. The ability to directly affect the spiking activity through the optical stimulation of synapses represents a transformative approach in neuromorphic engineering. Our optically tunable elements pave the way for advanced ANNs with highly efficient cognitive capabilities for emulating real brain dynamics.

Device Fabrication and Optoelectronic Measurements

ITO (40 nm), SiO_2_ (10 nm), and Ag (40 nm) were RF sputtered on m-glass at room temperature, while the FA_2_PbI_4_ film (600 nm) was spin-coated. The Pt NPs were deposited using the gas condensation terminated technique. For the recording of the LIF and synaptic responses, the HP 8116A Pulse Generator and the RIGOL MSO7071 digital oscilloscope were used. More details can be found in the Supporting Information file.

## Supplementary Material



## Notes

UV–vis, XPS, and UPS measurements for the perovskite film, energy band diagram of the perovskite film, I–V hysteresis patterns under consecutive cycling of the reference samples, the samples containing only Pt NPs and 5 nm SiO_2_, TEM measurements of the Pt NPs, dependence of the device resistance from the total area and environmental temperature, device-to-device distribution and stability of the devices over time, geometry of the simulated cell, details of the switching model, transient responses, temperature profiles, relaxation time, details of the segregation model, source data of the optical modulation process and bit precision, dark conditions spectrum, energy diagram configuration for the sample under consideration, experimental patterns of the STDP rule, electrical responses of the neuromorphic system, profiles of the input optical pulses, model parameter values, comparison of the memory and neuromorphic performance of various FAPI-based devices that have been reported in the literature. (PDF)
