# Supplementary material for: Low Power FA2PbI4/SiO2 Bilayer Memristors with Pt Nanoparticles Exhibiting Reconfigurable Synaptic and Neuron Properties for Compact Optoelectronic Neuromorphic Systems
Source: Nano Lett. 2025 Oct 6;25(41):14903–12. doi: 10.1021/acs.nanolett.5c03475 (PMC12532278; doi:10.1021/acs.nanolett.5c03475)
Supplement: Supplementary file 1 [file nl5c03475_si_001.pdf]

The Supporting Information is available free of charge on the ACS Publications website.

Supporting Information

Low Power FA_2PbI_4/SiO_2 Bilayer Memristors with Pt Nanoparticles Exhibiting Reconfigurable Synaptic and Neuron Properties for Compact Optoelectronic Neuromorphic Systems

Panagiotis Bousoulas, Spyros Orfanoudakis, Danai Spathi, Victoras Pagonis, Leonidas Tsetseris, Charalampos Tsioustas, Polychronis Tsipas, Athanassios G. Kontos, Thomas Stergiopoulos,* Dimitris Tsoukalas*

P. Bousoulas, S. Orfanoudakis, D. Spathi, V. Pagonis, L. Tsetseris, C. Tsioustas, A. G. Kontos, D. Tsoukalas

Department of Physics, School of Applied Mathematical and Physical Sciences

National Technical University of Athens

Iroon Polytechniou 9 Zographou, 15780, Greece

E-mail: panbous@mail.ntua.gr

S. Orfanoudakis, P. Tsipas, A. G. Kontos, T. Stergiopoulos

Institute of Nanoscience and Nanotechnology

NCSR Demokritos, 15341, Athens, Greece

E-mail: t.stergiopoulos@inn.demokritos.gr

S1. UV-vis measurements

Figure S1 presents the UV-vis spectrum of the FA_2PbI_4 film on an ITO substrate. The absorption spectrum displays a prominent shoulder at ~550 nm, which is characteristic of the excitonic absorption in FA_2PbI_4. At the same time, the absorption extends into the near-infrared beyond 700 nm, consistent with absorption from the 3D α-$FAPbI_3$ phase.

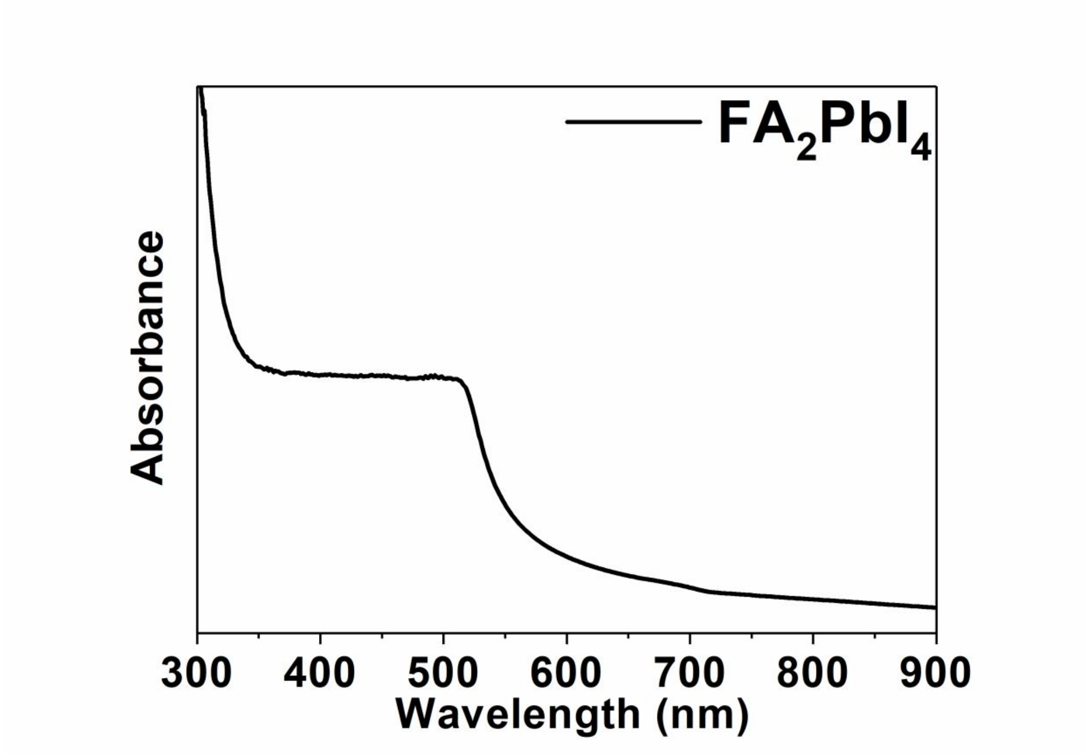

Figure S1. UV-vis spectrum of the ITO/FA_2PbI_4 film.

S2. XPS measurements

Figures S2a,b present the XPS spectra of the C1s and O1s cores, respectively.

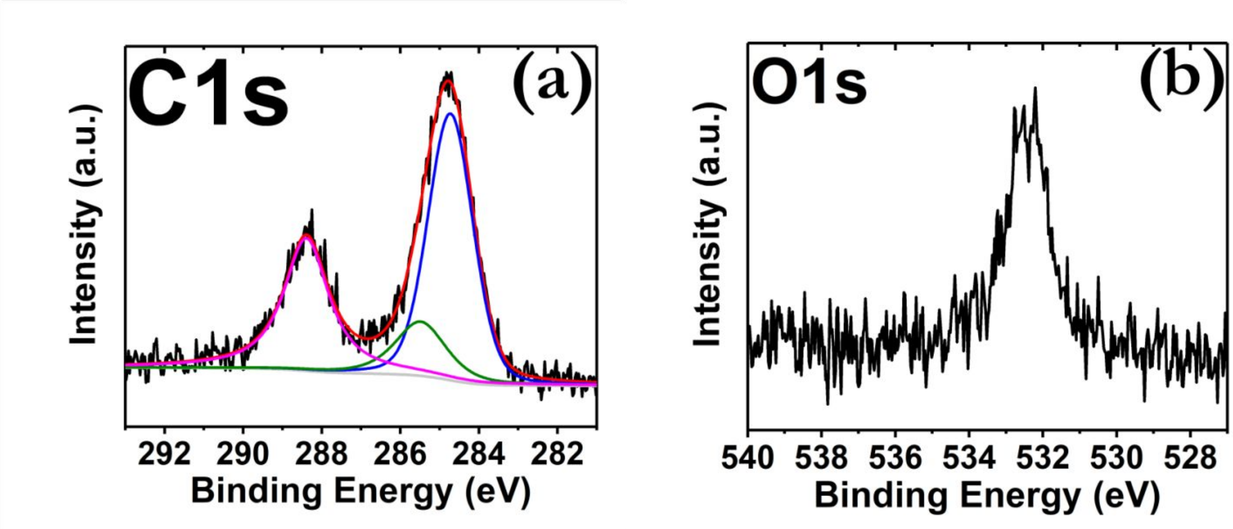

Figure S2. XPS spectra of the (a) C1s and (b) O1s spectra with the corresponding peaks after fitting analysis.

S3. UPS measurements

Figure S3 presents the Taus plots for the extraction of the work function of Fermi position level values.

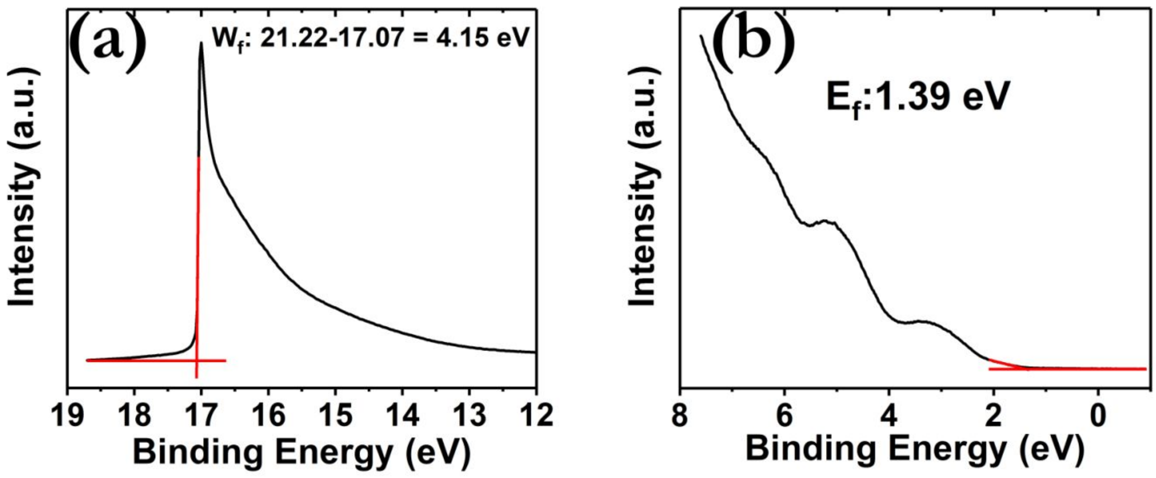

Figure S3. UPS spectra for the extraction of the (a) work function and (b) the Fermi level of the ITO/FA_2PbI_4 film.

S4. Energy band diagram extracted from UPS measurements

Figures S4a,b present the energy band diagram configuration and the respective calculations for the extraction of the $E_{FERMI,}$ E_{HOMO}, and E_{LUMO} levels.

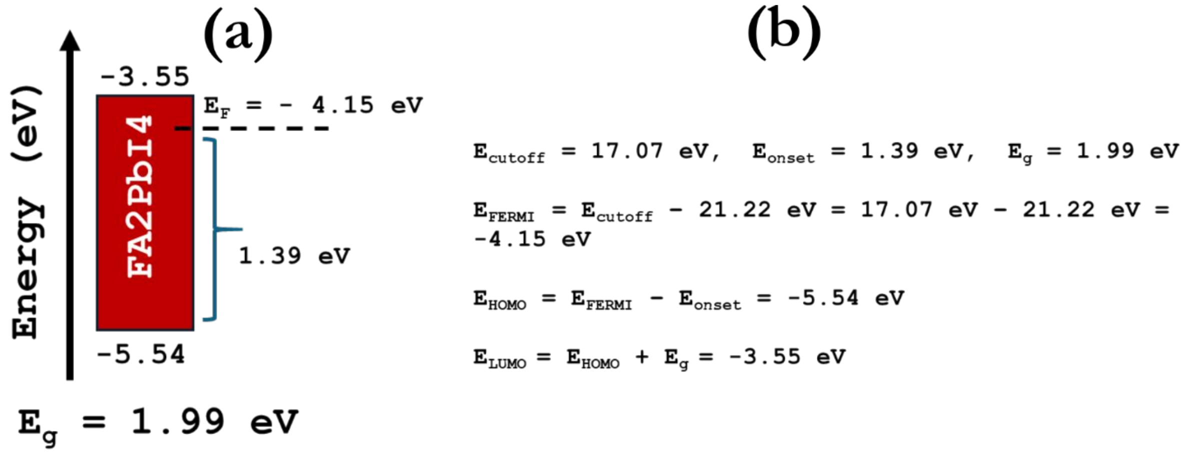

Figure S4. (a) Energy band diagram and the (b) respective calculations.

S5. I-V hysteresis patterns of the reference sample

Figure S5 illustrates the structure and the recorded hysteresis patterns of the reference sample, which exhibits a poor DC endurance behavior.

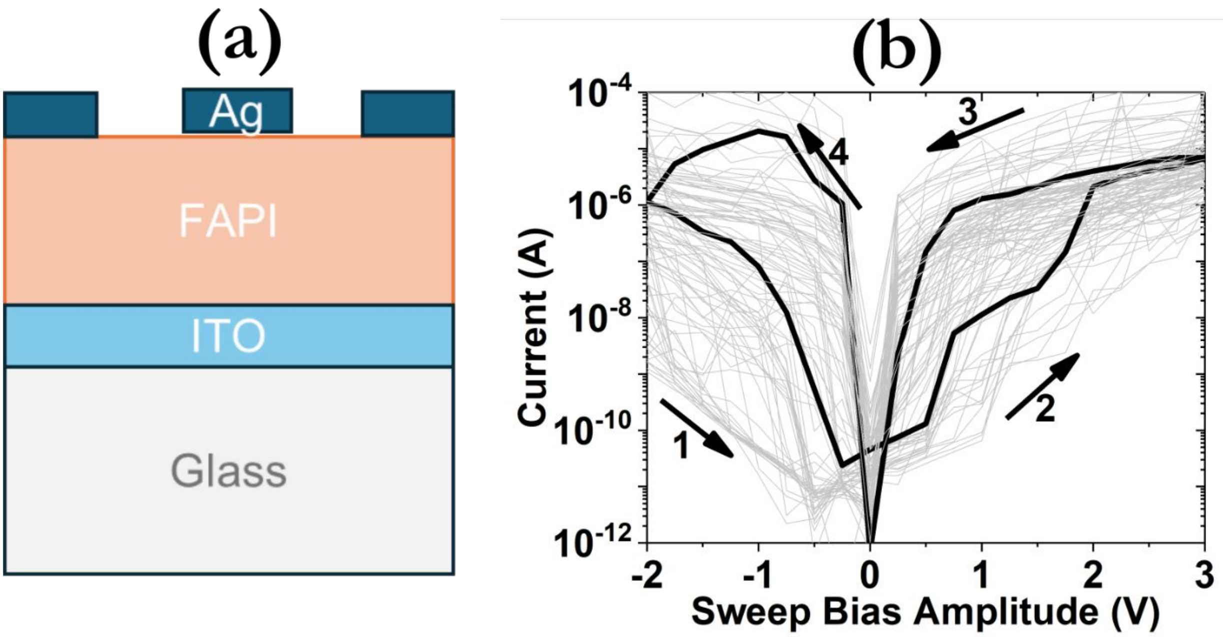

Figure S5. (a) Schematic illustration of the reference device configuration consisting only of the FA_2PbI_4 film. (b) I – V hysteresis patterns under the application of 100 DC endurance cycles with a constant I_{cc} of 10^{-4} A with a voltage scan rate of 200 mV/s. The numbers and arrows in the graph signify the switching direction. Similar hysteresis patterns were obtained by starting the voltage scans from 0 V to either positive or negative biases.

S6. I-V hysteresis patterns of the reference sample containing Pt NPs

Figure S6 illustrates the structure and the recorded hysteresis patterns of the reference sample containing a thin layer of Pt NPs. Sharper switching slopes were recorded, but the endurance pattern remains unsatisfactory.

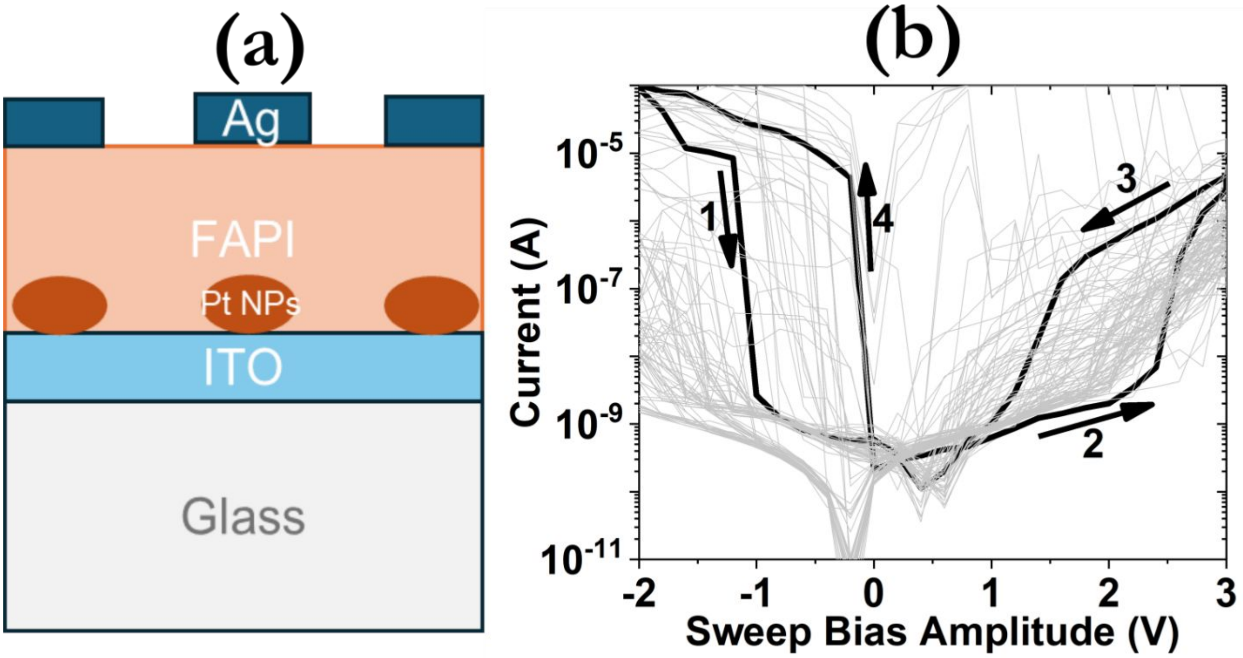

Figure S6. (a) Schematic illustration of the reference device configuration consisting only of the FA_2PbI_4 film and a thin layer of Pt NPs. (b) I – V hysteresis patterns under the application of 100 DC endurance cycles with a constant I_{cc} of 10^{-4} A with a voltage scan rate of 200 mV/s. The numbers and arrows in the graph signify the switching direction. Similar hysteresis patterns were obtained by starting the voltage scans from 0 V to either positive or negative biases.

S7. I-V hysteresis patterns of the reference sample containing Pt NPs and 5 nm SiO_2

Figure S7 illustrates the structure and the recorded hysteresis patterns of the reference sample containing a thin layer of Pt NPs and 5 nm of SiO_2. The DC endurance patterns exhibit an improved pattern with respect to the two previous cases (Figures S5 & S6), but some variations still exist.

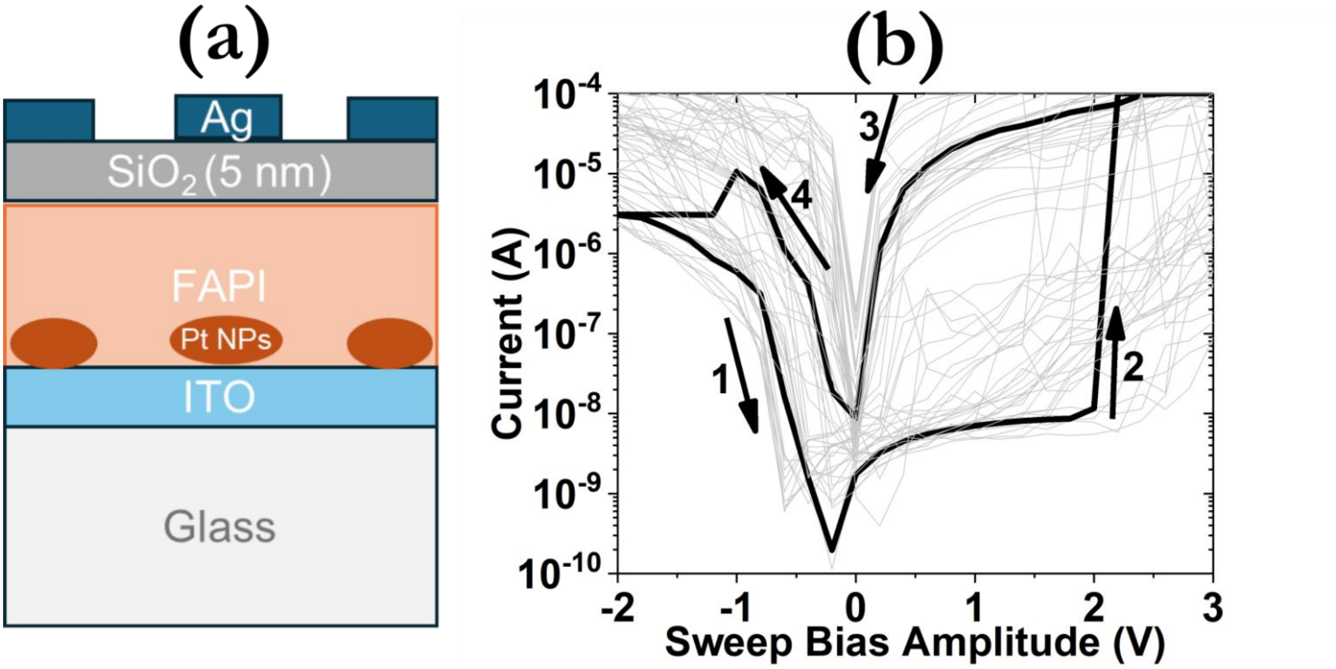

Figure S7. (a) Schematic illustration of the reference device configuration consisting only of the FA_2PbI_4 film and a thin layer of Pt NPs. (b) I – V hysteresis patterns under the application of 100 DC endurance cycles with a constant I_{cc} of 10^{-4} A with a voltage scan rate of 200 mV/s. The numbers and arrows in the graph signify the switching direction. Similar hysteresis patterns were obtained by starting the voltage scans from 0 V to either positive or negative biases.

S8. TEM measurements and nanoparticle generation

Figure S8a presents the TEM measurements and Figure S8b the corresponding histogram of their distribution, where their average size (~3 nm) and surface density (~2 x 10^{12} NPs/cm^2) can be seen. The nanoparticles (NPs) are generated by using the direct current (DC) magnetron sputtering method, followed by gas phase condensation. In a DC magnetron sputtering system (Figure S8c), the plasma state is induced by the inert gas being close to the target thanks to the magnetic field of the DC magnetron sputtering. The result is the formation of supersaturated vapors of the target containing its atoms. Typically, entering the agglomeration zone where the gas exists, the processes of condensation and nucleation take

place, resulting in the creation of a distribution of NPs with a variety of sizes. The nucleation of these small particles (granules) is followed by the growth of granules into larger particles (clusters). The development of swarms is strongly dependent on cross-sectional collisions (here the presence of inert gas becomes apparent). The growth and nucleation of the cluster are stopped as the cluster enters through a small hole in the filtration zone, where significantly lower pressure prevails. In this phase, the NPs that will be deposited on the substrate are formed. The deposition conditions affecting the surface density of NPs and their size distribution, are the temperature of the substrate, the deposition time, the strength of the deposition and the inert gas flow rate.

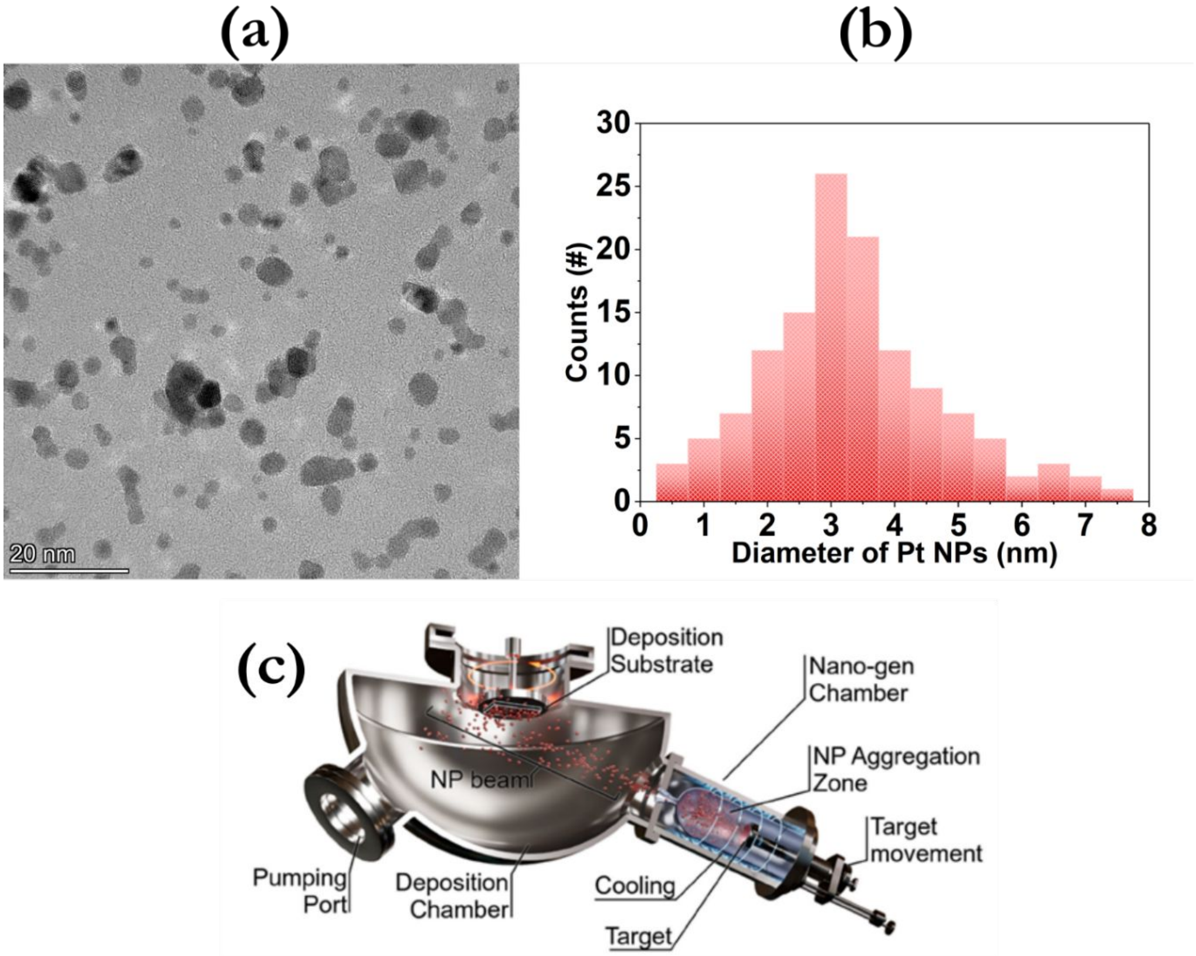

Figure S8. (a) TEM plan view image of the Pt NPs layer. The scale bar corresponds to 20 nm. (b) Histogram of the NPs' diameter. (c) Schematic illustration of the NPs generation process.

The formation and surface distribution of Pt NPs were optimized by systematically tuning the size of the aggregation zone, substrate temperature, deposition time, sputtering power, and argon gas flow rate during DC magnetron sputtering:

- **Size of the aggregation zone:** In this region, the nucleation of these small particles (granules) is followed by the growth of granules into larger particles (clusters). The development of swarms is strongly dependent on cross-sectional collisions. A size of 10 cm was selected for this zone.
- **Substrate Temperature:** Increasing the substrate temperature from room temperature up to 200 °C can promote the adatom mobility, leading to the formation of well-separated, uniformly distributed nanoparticles. At lower temperatures, Pt NPs tended to agglomerate, resulting in broader size distributions. A room temperature process was applied here since the deposition time was relatively small.
- **Deposition Time:** Short deposition times (<10 s) produced low surface coverage with sparsely distributed nanoparticles, while excessively long times (>180 s) resulted in nanoparticle coalescence. An optimized time window of 40 s was selected to achieve uniform coverage with controlled particle size.
- **Sputtering Power (Deposition Strength):** The application of a higher sputtering power (50–60 W) increased the nucleation rate, leading to smaller particle sizes but higher density. A moderate power of 50 W was found optimal for balancing nanoparticle density and size uniformity.
- **Inert Gas Flow Rate:** The argon flow rate controlled the plasma density and kinetic energy of sputtered Pt atoms. Lower flow rates (<15 sccm) yielded insufficient deposition, while very high flow rates (>60 sccm) caused particle coalescence due to enhanced scattering. An optimized flow rate of 40 sccm was selected.

Compared to conventional wet-chemical routes, the preparation of Pt NPs by DC magnetron sputtering offers several advantages:

1. **Purity of Nanoparticles:** Sputtering is a physical vapor deposition method that does not require chemical precursors, surfactants, or stabilizers, resulting in highly pure Pt NPs without surface contamination.
2. **Uniformity and Control:** It enables precise control over particle size, distribution, and surface coverage by tuning deposition parameters such as power, time, and working pressure.
3. **Direct Deposition on Substrates:** Pt NPs can be deposited directly onto device substrates without the need for transfer or additional surface functionalization, which is particularly advantageous for optoelectronic and neuromorphic devices.
4. **Scalability and Reproducibility:** Magnetron sputtering is an industry-compatible technique, enabling high reproducibility and uniform coating over large areas, which is beneficial for integration into large-scale device fabrication.

S9. Device area and temperature dependence

To explore the validity of the filamentary concept, devices with different areas of the TEs, in terms of dimensions, were fabricated. The results presented in Figure S9a suggest the total independence of the LRS from the device area, whereas the HRS seems to be affected. Therefore, it can be inferred that, according to the filamentary theory, the formation of one conducting filament is enough to bridge the two working electrodes and switch the device's conductance levels. In addition, a slight increase in the LRS state was observed during the I-V measurements at different temperatures (Figure S9b), implying its metallic nature.

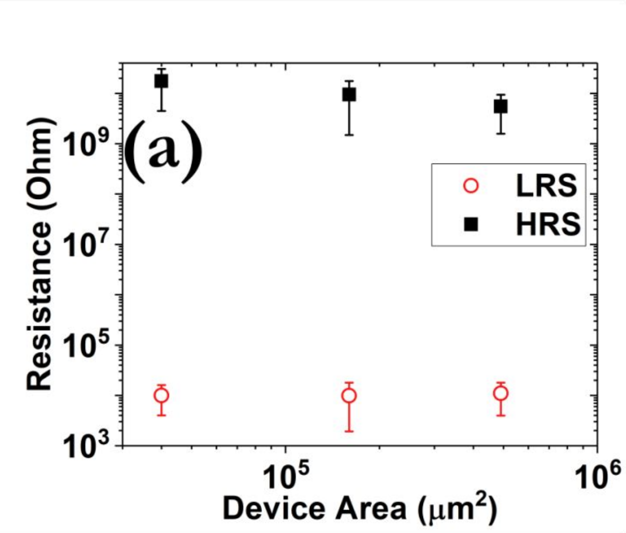

Figure S9. (a) Distribution of the HRS and LRS for all Samples (read voltage 1 V). (b) I-V hysteresis patterns recorded are different temperatures.

S10. Statistical variation and stability of the devices over time

The CDF plots concerning the device-to-device distribution are presented in Figure S10a. The data have been collected by measuring more than 200 devices a satisfactory σ/μ ratio was extracted for both HRS and LRS. Figure S10b depicts the measured hysteresis patterns of the FA2PBI4-based samples within a period of 4 months. As can be observed, the samples remained fully operational without recording any significant degradation of the switching characteristics and the memory window.

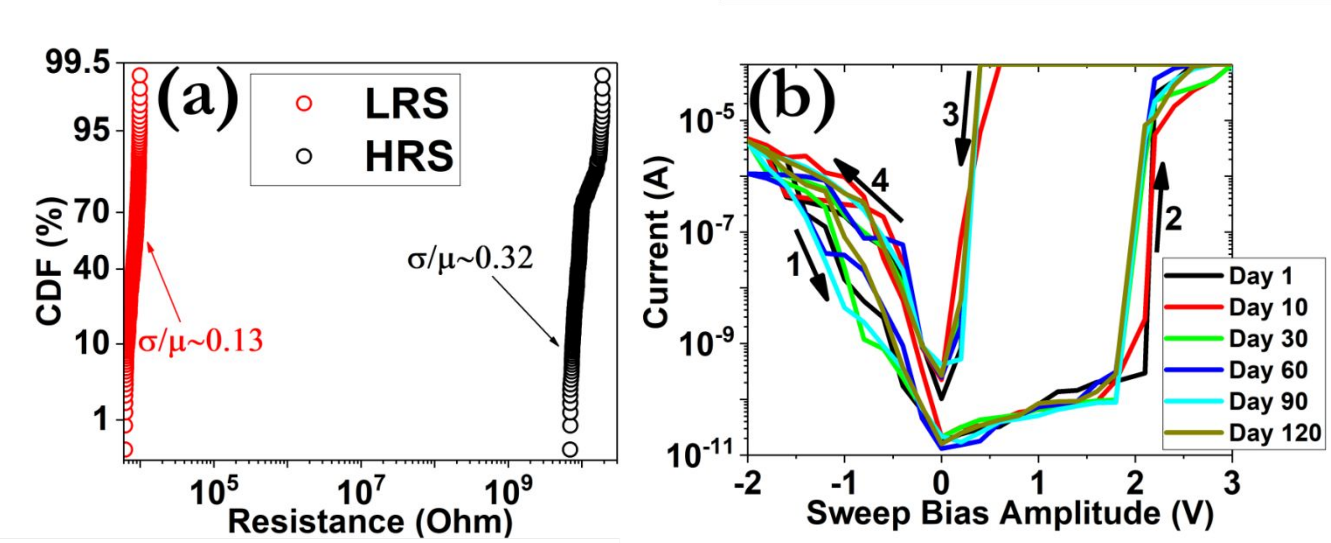

Figure S10. (a) Device-to-device distribution of the devices considered in this work (a read-out voltage of 1 V was used). (b) I-V hysteresis patterns at a period of 4 months.

S11. Geometry of the simulated cell

Figure S11 depicts the CF shape for the materials configurations studied in this work. The pulses were always applied on the TE, while a boundary condition of T = 300 K was enforced for the outermost surface of the electrodes, due to their considerably larger area with respect to the formed CF. The CF was set to be truncated - conical with a diameter of 20 nm at the position z = 650 nm, while a diameter of 6 nm was selected at the position z = 40 nm. This assumption was made to interpret the self-rectification properties of our devices, which could not be explained by solely taking into account the small difference (~0.1 eV) in the respective Schottky barriers between the operating electrodes and the switching material. Although this particular selection seems arbitrary, the simulated outcomes reveal good consistency with the experimental patterns. Moreover, the radial interference of the outmost surfaces of both FA_2PbI_4 and SiO_2 was set totally insulating.

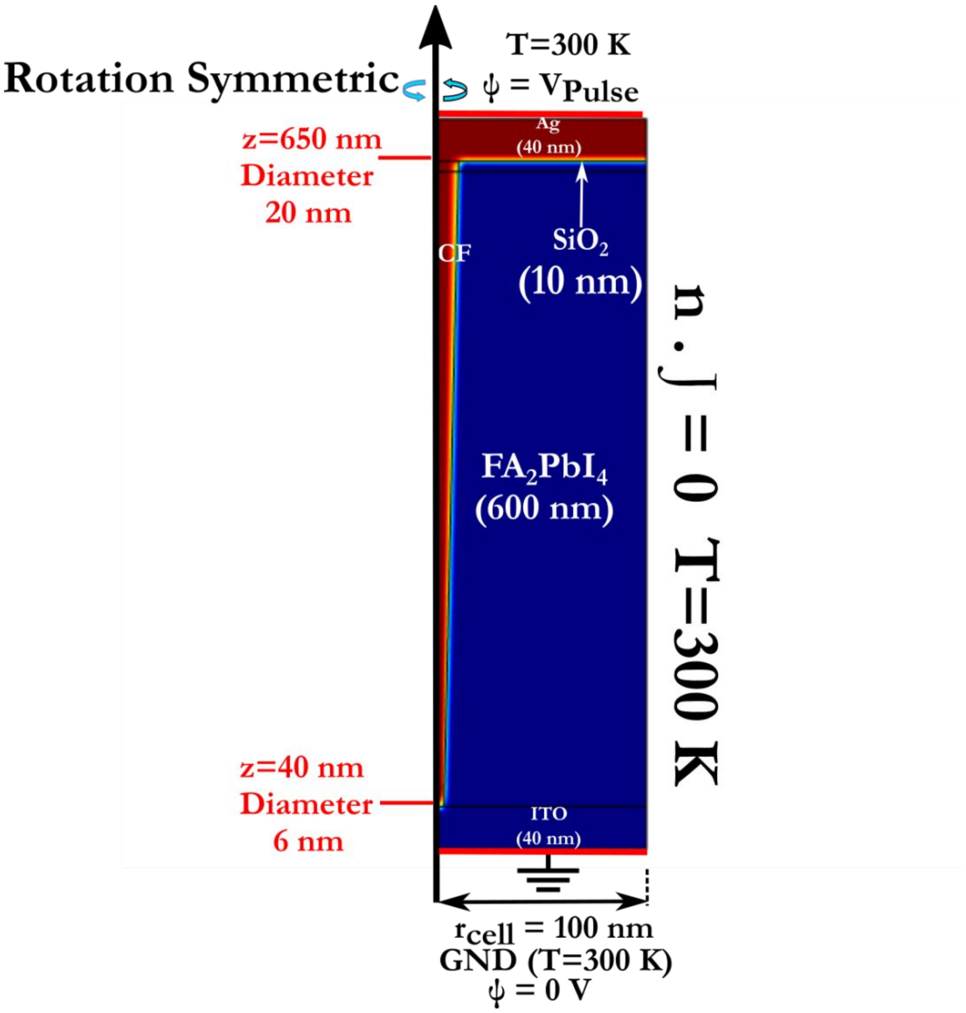

Figure S11. Cross-section representation of the simulated memory cell in 2D axisymmetric geometry with the respective boundary conditions for the FA2PBI4-based sample. The color denotes the distribution of the CF's diameter that consists of Ag NCs. The initial values of φ_B (this term represents the effective diameter of the CF at the interface with the BE) were chosen to reproduce the experimentally recorded operating current values.

S12. Switching model

The movement of silver cations, which are initially created through oxidation, within the halide perovskite and their reduction at the inert electrode can be accurately modelled considering the drift, diffusion, and thermos-diffusion fluxes. As a result, the two electrodes are connected with a metallic chain that induces the switching of the device's resistance. Thus, the memristive pattern can be simulated by calculating the CF's effective diameter (φ) by

solving the following three differential equations (drift-diffusion, current continuity, and Joule heating):[SI.1]

$$\frac{d\varphi}{dt}=\left.\frac{d\varphi}{dt}\right\|_{drift}+\left.\frac{d\varphi}{dt}\right\|_{diffusion}+\left.\frac{d\varphi}{dt}\right\|_{thermo-diffusion}=Ae^{-\frac{E_{drift}-aq\psi}{k_BT}}+B\varphi^{-1}e^{-\frac{E_{diff}}{k_BT}}-C\varphi^{-1}S\left(\frac{\partial T}{\partial r}+\frac{\partial T}{\partial z}\right) \quad (1)$$

$$\nabla\cdot\sigma\nabla\psi=0 \quad (2)$$

$$\rho_m C_p\frac{\partial T}{\partial t}=\nabla k_{th}\cdot\nabla T+\sigma\nabla\left|\psi\right|^2 \quad (3)$$

where k_B is the Boltzmann constant, T denotes the absolute temperature, α is the barrier lowering factor, E_{drift} and E_{diff} represent the energy barriers for ion hopping and diffusion, respectively, ψ refers to the electrical potential, σ is the electrical conductivity, E_S is the activation energy of thermophoresis, S is the Soret coefficient, k_{th} is the thermal conductivity, ρ_m is the mass density, C_p is specific heat, and A, B, and C are constants. The specific values of all parameters can be found in Table S1. The set of equations was solved simultaneously and self-consistently using a numerical solver (COMSOL). Figure S12a shows the distribution of the electrical conductivity and thermal conductivity functions from the effective diameter of the CF, while a correlation of the simulated CF dimensions at its thinnest part with the various applied I_{cc} values is presented in Figure S12b.

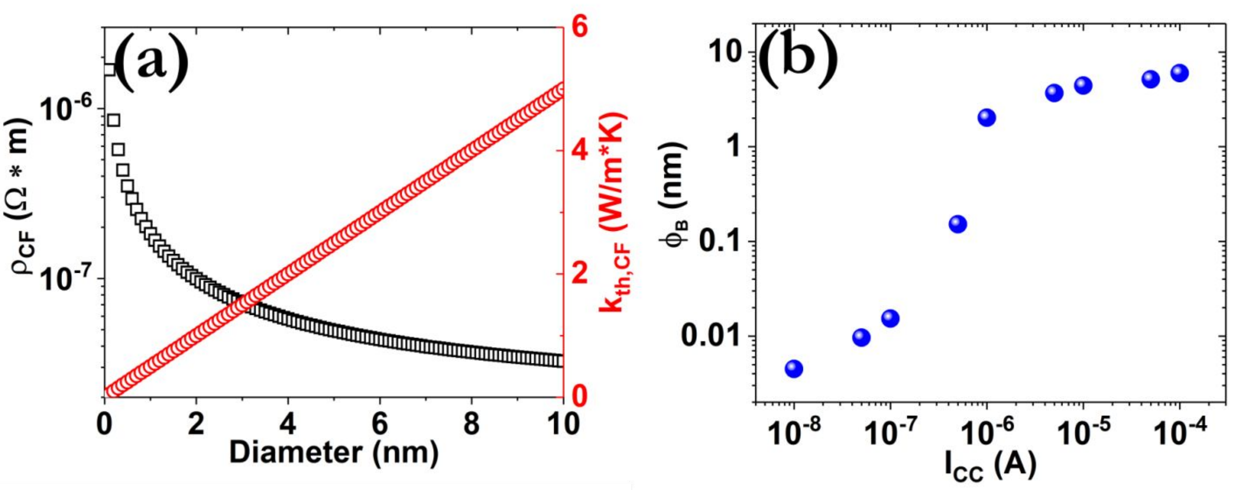

Figure S12. (a) Dependence of the electrical resistivity and the thermal conductivity as a function of φ for Ag-based CFs. (b) Dependence of the filament diameter from the applied I_{cc}.

S13. Simulated profiles for the SET transitions

The distribution profiles of the applied bias, effective diameter φ_B, and total resistance are presented in Figure S13. A triangular pulse with a rate of 10 mV/s was applied to induce the SET transition. At about 0.7 – 1 s, the calculated current values start to increase, demonstrating the onset of the former transition. For the applied I_{cc} of 1 μA, it can be seen that at t = 3 s, the effective diameter becomes equal to the respective value at t = 0 s, signifying the manifestation of a threshold switching phenomenon, whereas, for the bigger applied I_{cc}, no such effect takes place. The simulated profiles can smoothly emulate the respective DC experimental pattern, as well as the recorded transition slopes.

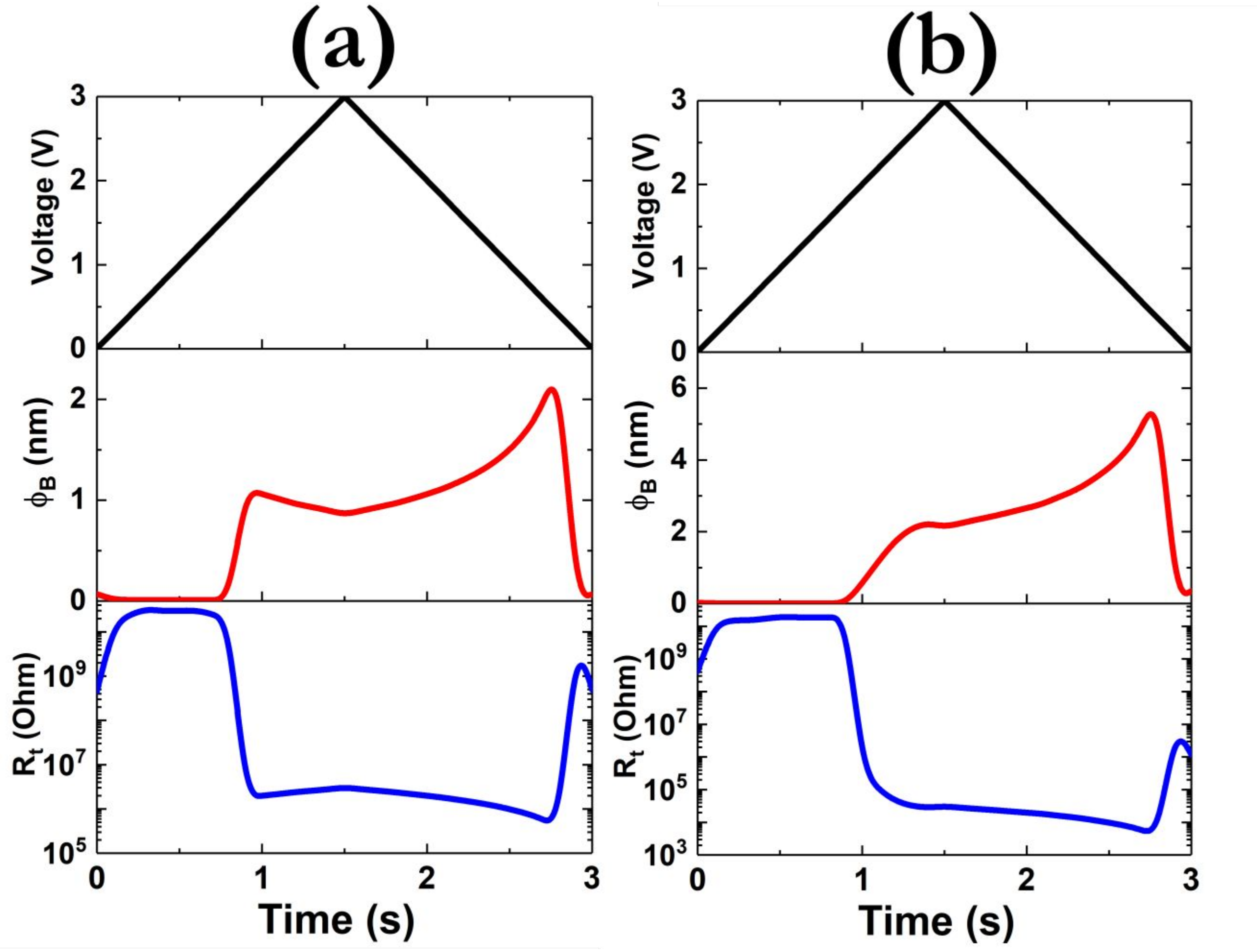

Figure S13. Distribution of the applied bias, diameter of the CF in the vicinity of the BE, and total resistance during the SET transition for I_{cc} values of (a) 1 μA and (b) 100 μA.

S14. Simulated profiles for the RESET transitions

Figure S14 presents the distribution of similar characteristics during the RESET process, which were presented above. Smaller CFs were used with respect to the application of a positive bias, in terms of the effective diameter distribution, to account for the self-rectification properties. The values of the diameters were selected to provide the respective operating current values of the experimental data. This is attained by choosing a suitable set of fitting parameters, as is reported in Table S1. There is also a physical explanation for the distribution of the effective CF's diameter near the BE. The development of local high temperatures decisively affects the bottom filament diameter. In other words, a higher local temperature leads to a smaller diameter of the CF during the RESET transition.

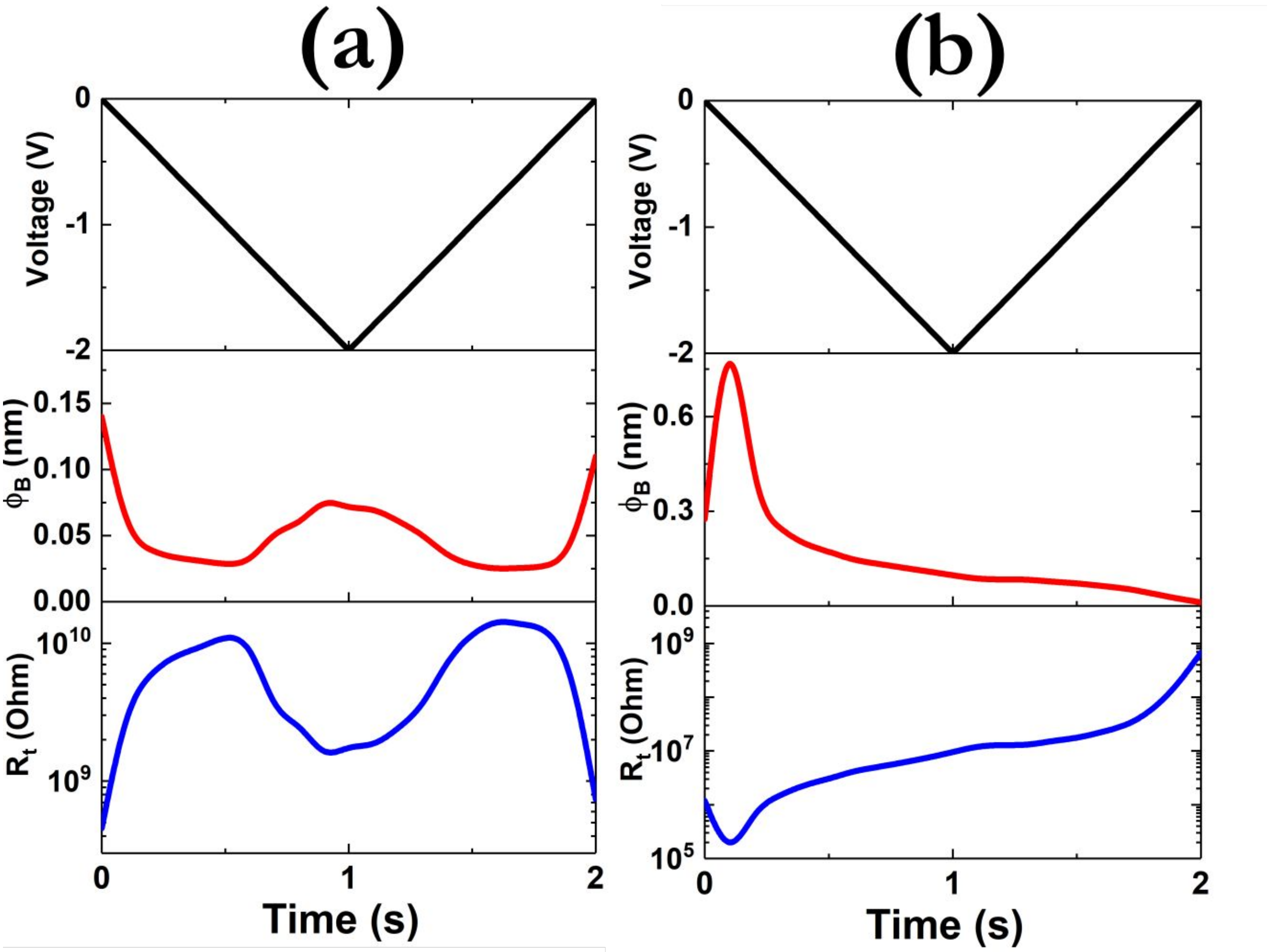

Figure S14. Distribution of the applied bias, diameter of the CF in the vicinity of the BE, and total resistance during the RESET transition for I_{cc} values of (a) 1 μA and (b) 100 μA.

S15. Transient responses

The transient operation of our devices can be also well captured by the proposed model, as can be observed from Figure S15. The symbol t_{SET} represents the time required to observe a change in the measured current (i.e., switching speed).

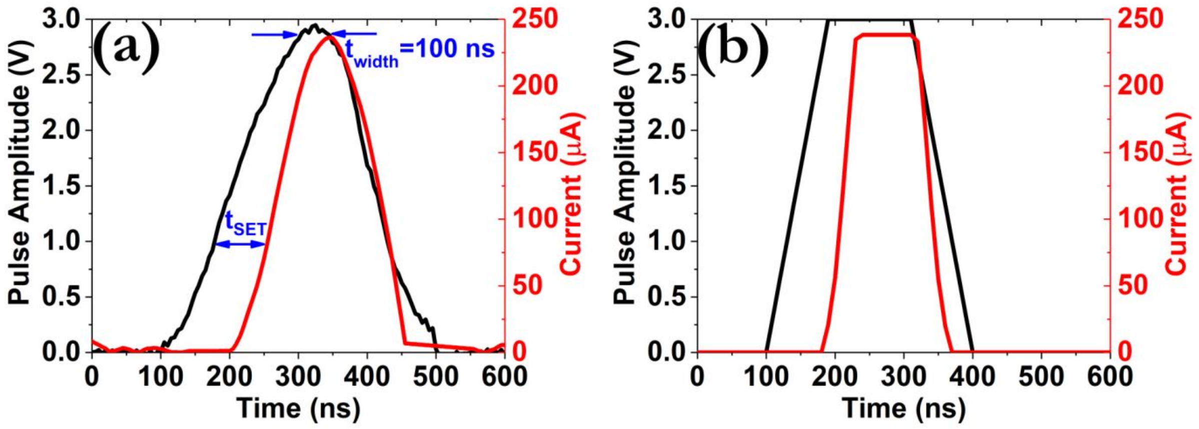

Figure S15. (a) Measured and (b) calculated pulsed I-V characteristics under the application of a square pulse with an amplitude of 3 V and a width of 100 ns.

S16. Temperature profile

Figure S16 depicts the formation and rupture procedures that take place within the device's active core. The CF is assumed to grow toward the anode, eventually leading to the formation of a continuous conductive path between the two electrodes. As a result, the device switches to the LRS due to the formation of a metallic bridge (Figure S16(a)). The application of a reverse voltage leads to the rupture of the filament. The filament eventually breaks, disrupting the conductive path (Figure S16(b)). The Joule heating effect plays a crucial role here considering that the current flowing through the filament generates heat, which can eventually weaken it. The generated heat could be as high as 780 K (Figure S16(c)), which could melt the respective constituents of the CF. Considering that during the manifestation of the volatile

switching mode, the calculated values of the φ_B are smaller than 2 nm, the generated heat suffices to locally melt the tip of the CF and induce the creation of a small gap. Hence, the device spontaneously reverts to the HRS.

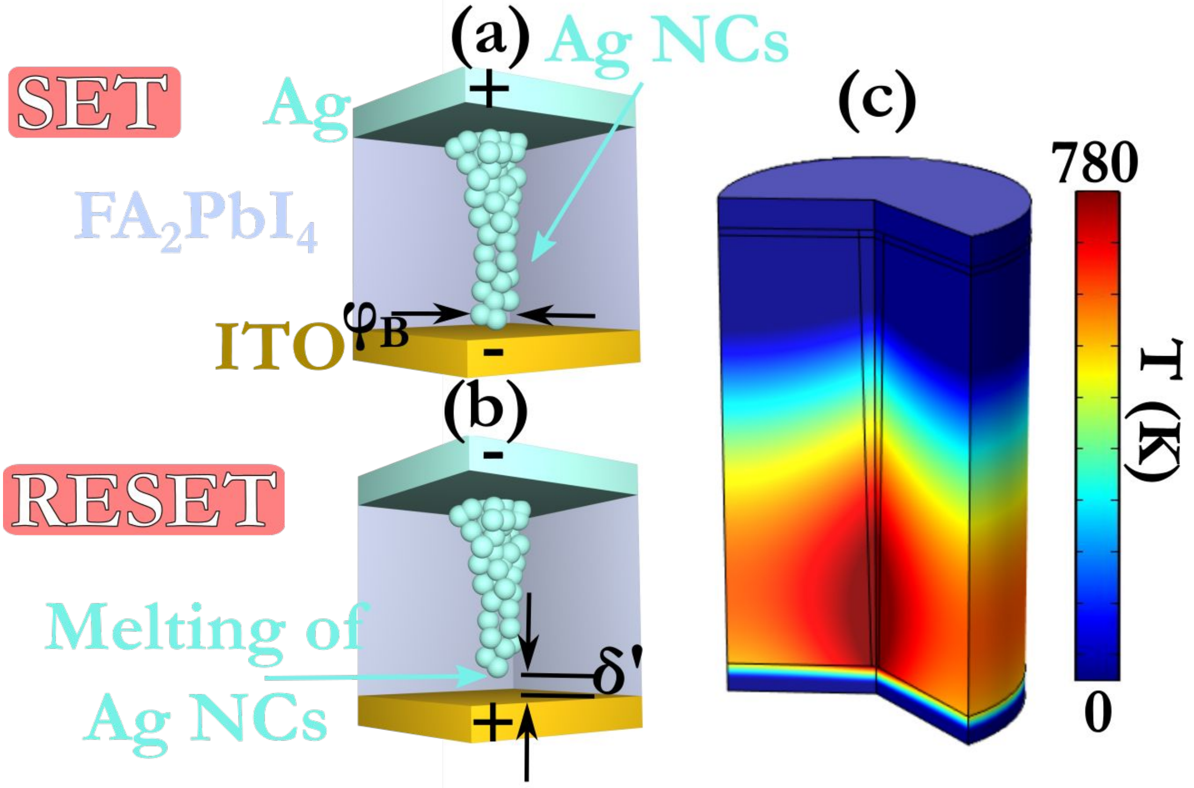

Figure S16. Schematic representation of the CF formation and annihilation processes during (a) SET and (b) RESET transitions. (c) 3D calculated maps of the localized temperature distribution at 1 V bias during switching direction 3.

S17. Relaxation time

Figure S17 presents the experimentally measured pulsed I-V characteristics during the application of the two-pulsing scheme. Even when the second monitoring pulse is enforced with a delay time of 100 ns, the memory cell is detected at the OFF-state, suggesting that the thermal effect plays a key role in shaping the switching pattern.

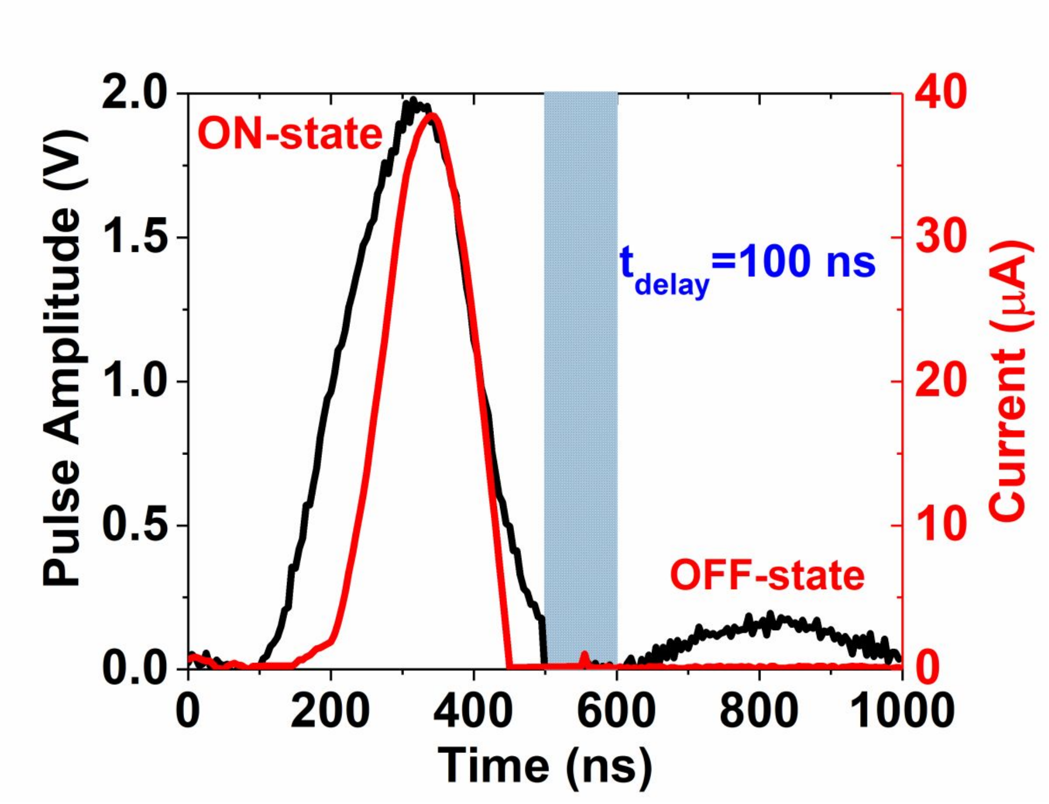

Figure S17. Pulsed I-V characteristics for the extraction of the relaxation time under the application of the two pulsing scheme protocol. The first triggering pulse has an amplitude of 2 V and a 100 ns width, whereas the second monitoring pulse has a lower amplitude of 200 mV and the same width as the previous pulse. The time delay between the two pulses is 100 ns.

S18. Segregation model

To examine the influence of phase segregation on the formation and rupture dynamics of conductive filaments, we modified our numerical model by incorporating an additive segregation term inspired by the Cahn–Hilliard equation. The Cahn–Hilliard formalism captures this behavior by introducing a chemical potential term derived from the free energy of mixing, which naturally accounts for the tendency of the system to minimize its Gibbs free energy. Specifically, the segregation flux can be expressed as proportional to the gradient of

the chemical potential, where the free energy contribution includes both local composition-dependent terms and interfacial energy penalties. The drift-diffusion equation of our model was modified as follows (Ref. 61-63 in the revised manuscript):

$$\frac{d\varphi}{dt} = \frac{d\varphi}{dt}\bigg\|_{drift} + \frac{d\varphi}{dt}\bigg\|_{diffusion} + \frac{d\varphi}{dt}\bigg\|_{thermo-diffusion} + \frac{d\varphi}{dt}\bigg\|_{segregation} =$$

$$Ae^{-\frac{E_{drift}-aq\psi}{k_B T}} + B\varphi^{-1}e^{-\frac{E_{diff}}{k_B T}} - C\varphi^{-1}S\left(\frac{\partial T}{\partial r}+\frac{\partial T}{\partial z}\right) - K_{seg}S^{-1}(\varphi)\mu_{seg}(c) \qquad \text{(S1)}$$

where K_{seg} is the coupling coefficient for phase-segregation-induced shrinkage, S(φ) is the area factor, and $\mu_{seg}(c)$ is the segregation chemical potential. A sidewall-limited exchange mechanism was selected for the area factor (S(φ) = πφ), which indicates that segregation drains halide ions along the CF sidewalls. The segregation chemical potential can be estimated as follows (derived from a regular-solution free energy with a gradient penalty collapsed to a 0D proxy):

$$\mu_{seg}(c) = k_B T \ln\left(\frac{c}{1-c}\right) + \Omega_{\text{int}}(1-2c) + \kappa\left(\frac{c-\bar{c}}{l^2}\right) \qquad \text{(S2)}$$

where Ω_{int} is the interaction energy between halide ions and controls the demixing tendency, c is the local halide fraction/order parameter (0–1), κ is the gradient energy coefficient, l is the characteristic segregation length scale, and $\bar{c}$ is the equilibrium halide concentration. A minus sign of $\mu_{seg}(c)$ was selected to highlight that this mechanism opposes the CF's diameter growth (demixing drive). Typical values for the associated parameters in the segregation flux are provided in Table S2.

From our calculations, a relatively small segregation flux ~10^{-17} m/s was extracted for K_{seg} = 10^{15} $m^{-2}\cdot s^{-1}\cdot eV^{-1}$, while the flux was increased to ~10^{-11} m/s and ~10^{-7} m/s for K_{seg} = 10^{20} and 10^{22} $m^{-2}\cdot s^{-1}\cdot eV^{-1}$, respectively. Only in the latter case, the segregation flux becomes comparable with the drift and diffusion fluxes. The simulated results are shown in Figure S18, suggesting a transition from bipolar to threshold switching for K_{seg} = 10^{22} $m^{-2}\cdot s^{-1}\cdot eV^{-1}$ and a

bigger V_{SET}. The latter effects are anticipated since the phase segregation flux together with the thermophoresis flux synergistically contribute to the dissolution of the CF.

The segregation coefficient K_{seg} in the Cahn–Hilliard framework represents the interfacial energy penalty associated with compositional gradients during phase segregation in mixed-halide perovskites. Physically, K_{seg} quantifies the energetic cost of forming interfaces between regions of different halide compositions (e.g., I-rich and Br-rich domains). However, the value of $K_{seg} = 10^{22}$ $m^{-2}\cdot s^{-1}\cdot eV^{-1}$ is extremely big for mixed halide perovskites. In particular, for mixed-halide lead perovskites such as $MAPb(Br_xI_{1-x})_3$, the experimental fits and phase-field simulations often report K_{seg} values of about 10^{18} $m^{-2}\cdot s^{-1}\cdot eV^{-1}$. Providing a precise, experimentally verified value for K_{seg} in FA_2PbI_4 is challenging because it is a 2D perovskite and its specific halide segregation kinetics are less commonly quantified in the same way as 3D mixed-halide analogs (e.g., $MAPb(I,Br)_3$). The relevant phase instability for this material is often related to hydration or transition to non-perovskite phases.

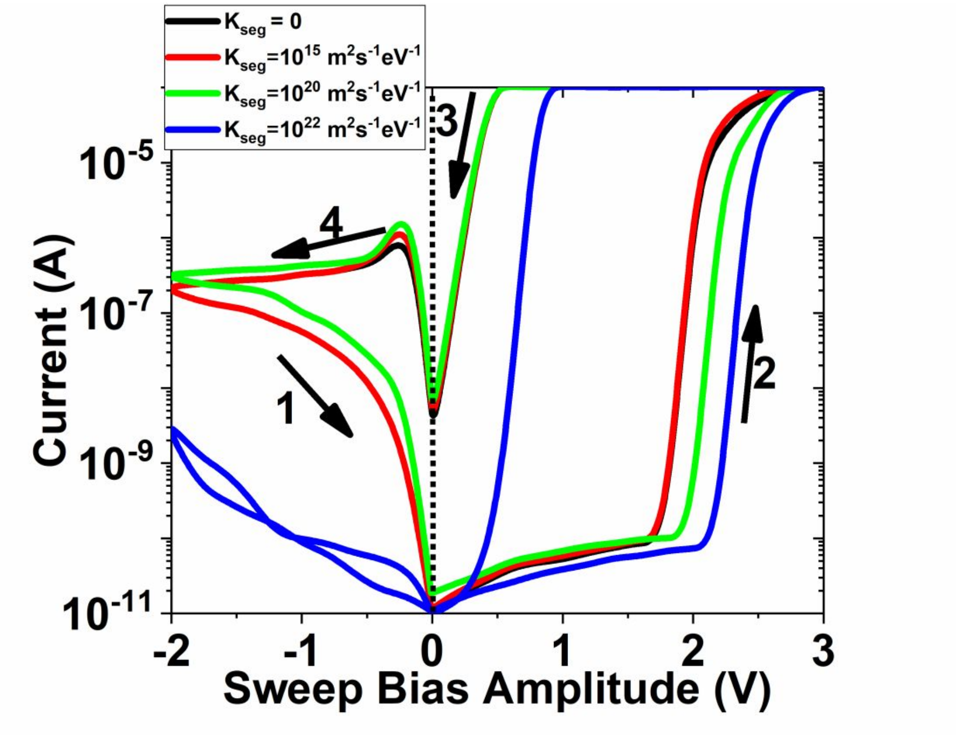

Figure S18. Calculated I-V characteristics by taking into account the influence of the phase segregation effect.

S19. Source data of the optical modulation process and bit precision

The source data during the optical modulation process are provided in Figure S19a, while Figure S19b depicts the bit precision results and their retention patterns. An exponential fitting model was applied to interpret the conductance modulation patterns:

$$G_{norm,p} = \frac{1}{a_p}\ln(we^{a_p} + (1-w)) + G_{\max} \tag{S3}$$

$$G_{norm,d} = (1-\frac{1}{a_d})\ln(we^{a_d} + (1-w)) + G_{\min} \tag{S4}$$

where α_p and α_d are the curvatures of the potentiation and depression curves, respectively, and w is defined as the ratio t/t_{max} and t is the pulse number. Hence, it can be quickly ascertained that relatively large values of the coefficients α_p and α_d indicate a high degree of nonlinearity of the conductance modulation while in the opposite case (α_p and $\alpha_d \rightarrow 0$) the corresponding curves become perfect straight lines.

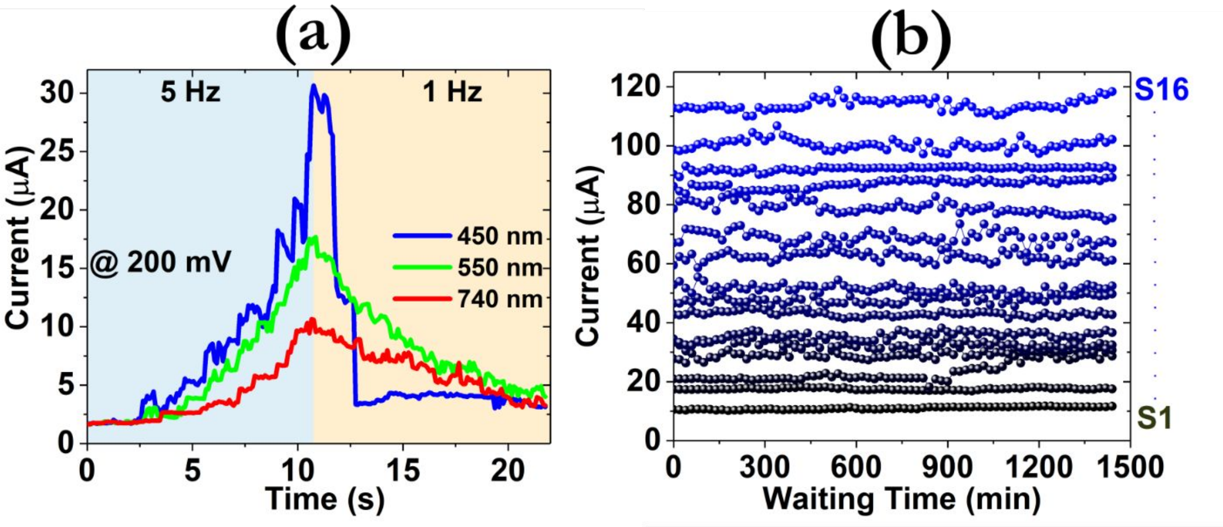

Figure S19. (a) Continuous modulation of the conductance states during consecutive device operation under light irradiation with various wavelengths and different frequencies at a constant light intensity of 1.4 mW/cm^2. (b) Retention results of 16 different current states after the application of an increasing number of optical pulses at 450 nm. All EPSCs responses were recorded at a read-out voltage of 200 mV.

S20. Dark conditions spectrum

To distinguish purely photonic effects from possible thermal or electrical artifacts in our optically driven synaptic modulation experiments, measurements under identical electrical conditions but in the absence of illumination were performed (Figure S20). The devices exhibited negligible conductance changes and the recorded current values were 4 orders of magnitude smaller than the illumination case, confirming that the observed modulation arises predominantly from photo induced carrier dynamics rather than electrical drift alone.

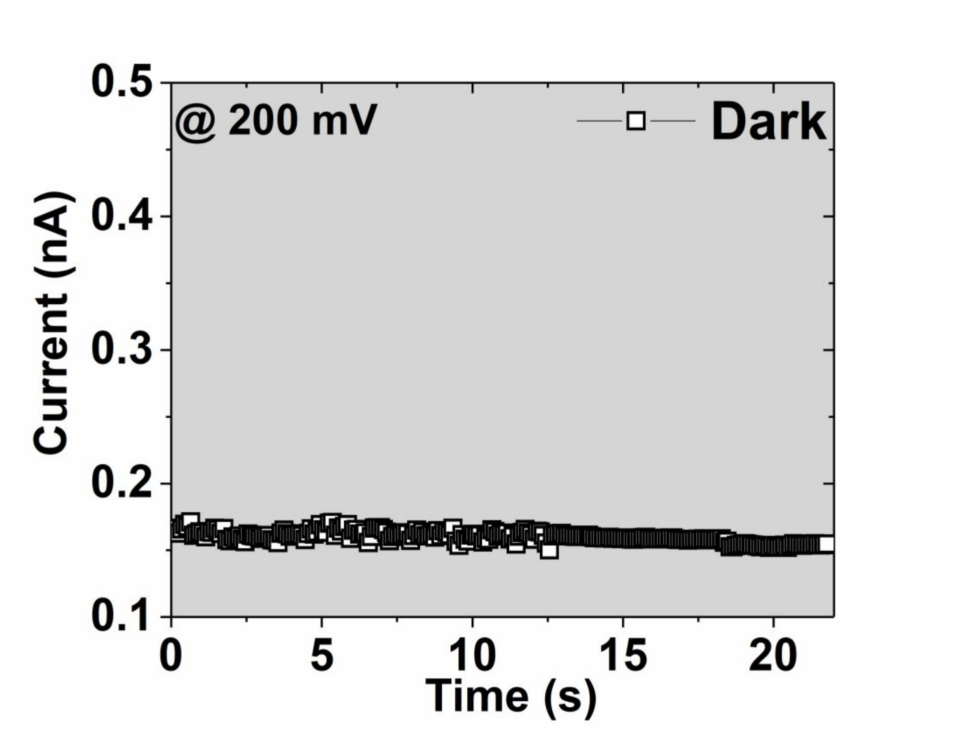

Figure S20. Current spectrum after the application of solely electrical read out pulses of 200 mV / 100 ms.

S21. Energy band diagram

The energy diagram configuration for the sample under consideration is displayed in Figure S21, where the whole stack is taken into consideration. A barrier with a height of 2.8 eV is formed between the SiO_2 and the FA_2PbI_4 layers, which could interpret the relatively big SET

voltages of the respective samples. Under high-frequency light irradiation, a large number of electron–hole pairs are generated within the FA_2PbI_4 perovskite layer. The photogenerated electrons tend to accumulate near the SiO_2/ FA_2PbI_4 interface, leading to a local increase in negative charge density. This charge redistribution induces downward band bending in the FA_2PbI_4 conduction band near the interface, effectively reducing the effective barrier height for electron injection into the SiO_2 layer.

The band bending occurs due to the establishment of a photoinduced interfacial electric field, which modifies the energy alignment between the conduction band minimum of FA_2PbI_4 and the conduction band of SiO_2, thus facilitating thermionic emission and tunneling processes. As a result, electron transfer toward the SiO_2 layer becomes more efficient under high-frequency illumination. In contrast, when the devices are exposed to low-frequency light pulses, fewer electron–hole pairs are generated, leading to weaker charge accumulation and a less pronounced modification of the interfacial energy landscape. Consequently, the effective barrier height remains closer to its equilibrium value, and the enhancement of electron transport is significantly reduced.

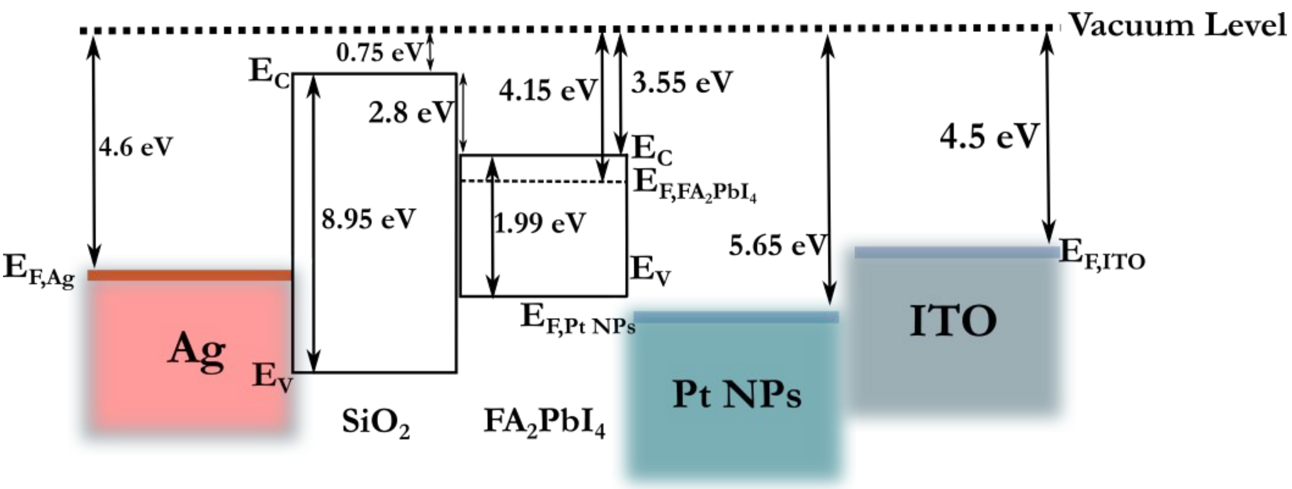

Figure S21. Energy band diagram of the various isolated materials under thermal equilibrium conditions, indicating the relative positions of the respective Fermi levels.

S22. STDP

Figure S22 depicts the fitting results for all illumination sources considered in this work. If a presynaptic neuron fires and then a postsynaptic neuron fires shortly afterward (Δt>0), the synaptic connection is increased, while in the opposite case, the synaptic connection is reduced. The synaptic weights were fitted through the STDP learning rule:

$$\Delta w(\Delta t) = \begin{cases} -w_{-}\exp\left(\frac{\Delta t}{\tau_{post}}\right), & \Delta t < 0 \\ w_{+}\exp\left(\frac{\Delta t}{\tau_{pre}}\right), & \Delta t \geq 0 \end{cases} \quad \text{(S5)}$$

where w_-, w_+, τ_{post}, and τ_{pre} are fitting constants.

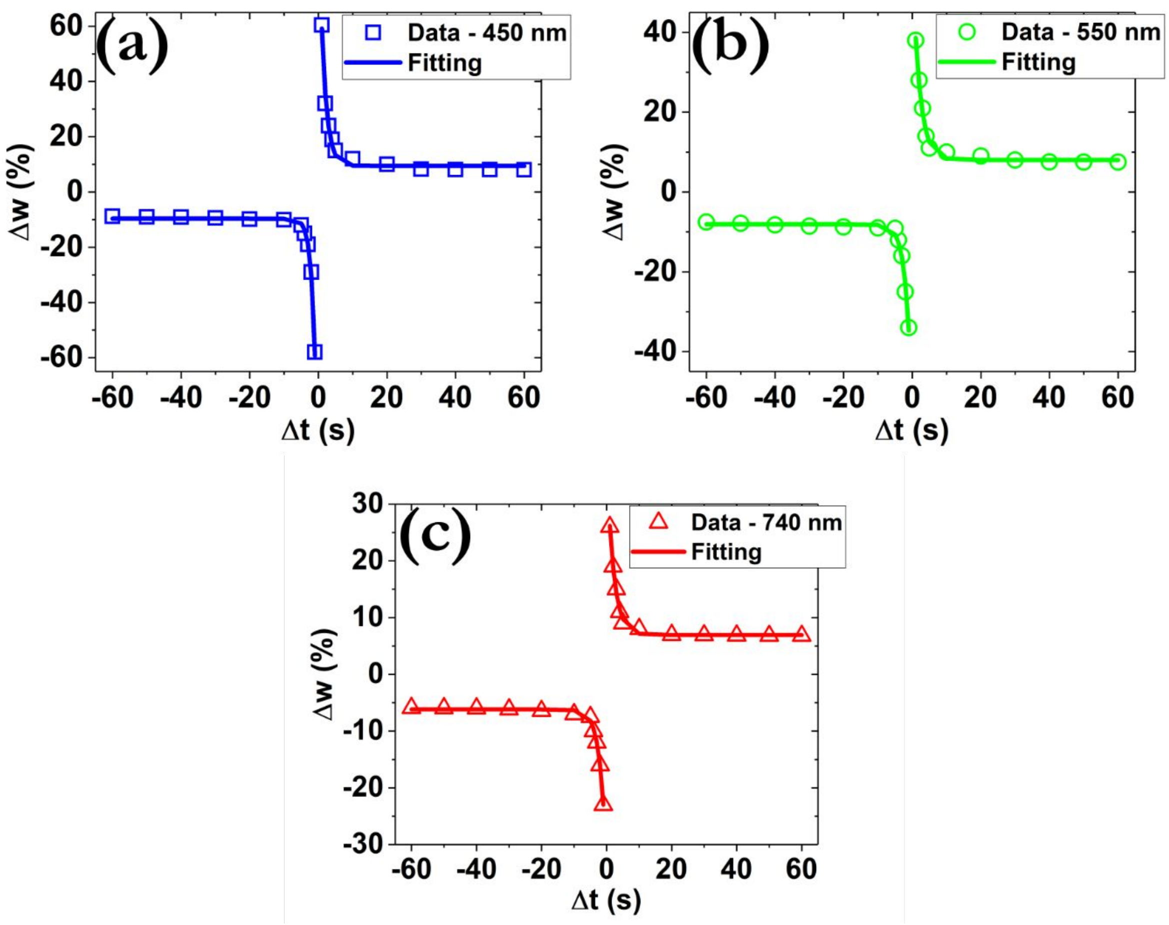

Figure S22. STDP pattern during the application of a nonoverlapping pulsing scheme under illumination with (a) 450 nm, (b) 550 nm, and (c) 740 nm optical pulses. The amplitude of the posts-spike electrical pulse was -1 V and a range of 0 – 60 s was used for the delay time. The

read-out process was conducted by applying square pulses with 200 mV amplitude and 100 ms width.

S23. Paired pulse facilitation (PPF) effect

The distribution of the facilitation process was also described using the following equation:

$$PPF = C_1 e^{\left(-\frac{t}{\tau_1}\right)} + C_2 e^{\left(-\frac{t}{\tau_2}\right)} \quad \text{(S6)}$$

where C_1 and C_2 are fitting constants and τ_1 and τ_2 represent the relaxation times of this effect.

From our analysis, the values of 1.03 and 4.22 s were estimated for the τ_1 and τ_2 times, respectively, which are similar to the response times of the biological synapses.

S24. Electrical responses of the neuromorphic system

Figure S24 depicts the responses of the artificial neuromorphic after the application of only electrical pulses. As can be seen, no spikes can be detected when the system is not irradiated since the accumulated V_s is smaller than the threshold voltage of the volatile memristor.

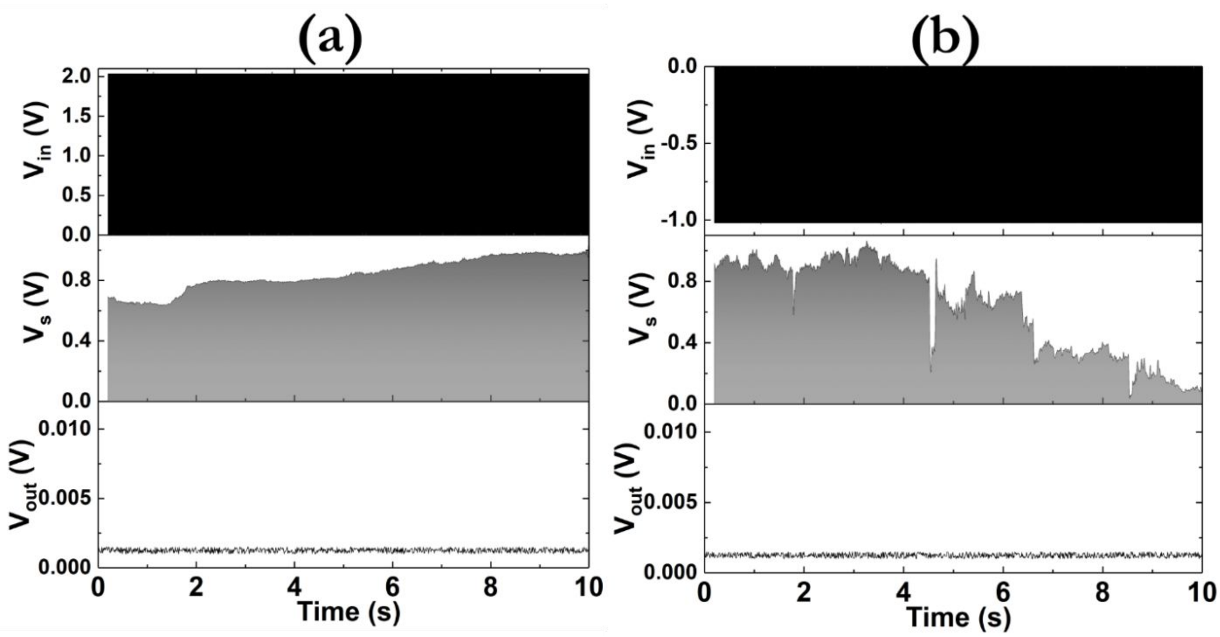

Figure S24. Experimental results of the evolution with time of the input voltage (V_{in} - black line), the voltage drop across the synapse (V_s), and the voltage at the output of the circuit (V_{out}) during the (a) potentiation and (b) depression processes. The amplitude of the input voltage was 2 V for the potentiation and -1 V for the depression processes, respectively, while the applied frequency was always 1 kHz.

S25. Profiles of the input optical pulses

The profiles of the applied optical pulses induced by the pulse generator are illustrated in Figure S25. The high optical frequency was used for the optical potentiation and the lower for the optical depression processes.

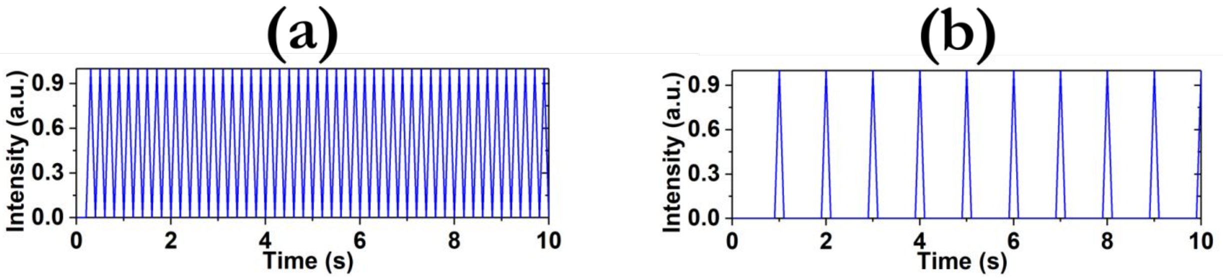

Figure S25. Profiles of the applied optical pulses with a constant width of 100 ms and frequency of (a) 5 Hz and (b) 1 Hz.

Table S1. Model parameters values.

Parameter	Positive Bias	Negative Bias
E_{drift}	FA_2PbI_4: 1.3 eV<br>SiO_2: 1.1 eV	
E_{diff}	FA_2PbI_4: 1.6 eV<br>SiO_2: 1.2 eV	
E_s	3 eV	
α	FA_2PbI_4: 0.2<br>SiO_2: 0.29	FA_2PbI_4: 0.85<br>SiO_2: 0.79
A	FA_2PbI_4: 5×10^{-4} ms^{-1}<br>SiO_2: 1×10^{-3} ms^{-1}	FA_2PbI_4:1×10^{-6} ms^{-1}<br>SiO_2: 3×10^{-5} ms^{-1}
B	FA_2PbI_4: 2×10^{-12} m^2s^{-1}<br>SiO_2: 1×10^{-11} m^2s^{-1}	FA_2PbI_4: 5×10^{-13} m^2s^{-1}<br>SiO_2: 2×10^{-13} m^2s^{-1}
C	FA_2PbI_4: 2×10^{-26} m^3s^{-1}<br>SiO_2: 1×10^{-26} m^3s^{-1}	FA_2PbI_4: 3×10^{-25} m^3s^{-1}<br>SiO_2: 1×10^{-25} m^3s^{-1}
$\rho_{bulk,m}$	Ag: 1.6×10^{-8} Ωm<br>ITO: 5×10^{-7} Ωm<br>Pt NPs: 2×10^{-7} Ωm	

$\rho_{bulk,FA2PbI4}$	1×10^4 Ωm
$\rho_{bulk,SiO2}$	1×10^3 Ωm
p	0.5
λ	40 nm
γ	5×10^{-8} mV^{-1}
$k_{th,FA2PbI4}$	5 $Wm^{-1}K^{-1}$
$k_{th,SiO2}$	10 $Wm^{-1}K^{-1}$
$k_{th,Ag}$	398 $Wm^{-1}K^{-1}$
$k_{th,ITO}$	4 $Wm^{-1}K^{-1}$
$k_{th,Pt\ NPs}$	0.1 $Wm^{-1}K^{-1}$
m	Ag: 5×10^8 $Wm^{-2}K^{-1}$
ρ_m	Ag: 10.497 kgm^{-3}<br>Pt NPs: 21.447 kgm^{-3}<br>ITO: 7200 kgm^{-3}<br>SiO_2: 2270 kgm^{-3}<br>FA_2PbI_4: 100 kgm^{-3}
C_p	Ag: 238 $Jkg^{-1}K^{-1}$<br>Pt NPs: 125 $Jkg^{-1}K^{-1}$<br>ITO: 341 $Jkg^{-1}K^{-1}$<br>SiO_2: 133 $Jkg^{-1}K^{-1}$<br>FA_2PbI_4: 110 $Jkg^{-1}K^{-1}$

Table S2. Model parameter values of the phase segregation effect.

Parameter	Typical Values	Role in the model
c	0.2 – 0.8	Fractional iodide content
$\bar{c}$	0.5	Reference composition around which segregation occurs
Ω_{int}	0.05 – 0.2 eV	Determines miscibility; larger values lead to stronger phase separation tendency
ϰ	1×10^{-10} $eV \cdot m^2$	Penalizes sharp composition gradients
l	1 – 10 nm	Defines spatial scale of composition modulation
K_{seg}	10^{15}–10^{22} $m^2 \cdot s^{-1} \cdot eV^{-1}$	Quantifies strength of phase-segregation effect on filament growth/shrinkage

Table S3. Comparison in the performance of various FAPI-based devices that have been reported in the literature.

Material	Switching Voltages (SET/RESET)	Switching Ratio	DC Endurance	AC Endurance	Non-volatile /Volatile	Ref.
$MAFAPbI_3$	0.35/-0.5 V	10^5	491	-	Non-volatile	[45]
$FAMAPbI_3$	1/-3 V	10^2	-	-	Non-volatile	[46]
δ-$FAPbI_3/SnO_2$	0.3/-1.5 V	10^2	3	10^3	Non-	[47]

					volatile	
$FAPbI_3$	0.2/-0.2 V	10^6	20	1200	Non-volatile	[48]
$FAPbI_3$	0.2/-0.2 V	10^6	5	2000	Non-volatile	[49]
OAI-$FAPbI_3$	-	-	-	-	-	[50]
PMMA-$FAPbI_3$	0.5/-0.5 V	10	5	250	Non-volatile	[51]
**SiO_2-FA_2PbI_4**	**1.7/-0.2 V**	**10^6**	**100**	**10^9**	**Both**	**This work**

Table S4. Comparison in the performance of various neuromorphic computing systems that have been reported in the literature.

Material	Synaptic Weight Precision	Linearity (a_p/a_d)	Retention	Energy/ weight change	Energy/ spike	Neuron jitter	Ref.
MoS_2-hBN	2 bit	-	100 s	~4 nJ	37.5 nW	1 – 10 ms	[6]
$HfAlO_x$	2.5 bit	-	600 s	~200 nJ	1.9 fJ	1 – 5 s	[7]
$VO_x/HfWO_x$	6 bit	0.04/0.11	-	~1 μJ	-	1 – 10 μs	[8]
HfO_2/NiO	-	-	-	~10 nJ	-	1 – 10 ms	[9]
$CsPbBr_3$	2.5 bit	-	-	~10 μJ	-	1 – 10 ms	[10]
NiO	-	-	800 s	~100 nJ	-	-	[11]
SiC	-	-	-	~10 nJ	-	1 – 10 ms	[12]
Li_xSiO_y and SiO_2:Ag	-	-	-	~1 μJ	-	-	[13]
MoS_2	3 bit	-	25 s	0.05 aJ	-	-	[SI.2]
h-BN	10 bit		10^4 s	100 aJ	-	-	[SI.3]
**SiO_2-FA_2PbI_4**	**4 bit**	**3.1/1.1**	**1400 s**	**1 nJ**	**10 fJ**	**1-10 ms**	**This work**

Experimental Details

Synthesis of FA_2PbI_4. Lead iodide (PbI_2, 99.99%) was supplied by TCI EUROPE N.V., Formamidinium iodide (FAI, 99.99%), was acquired from Greatcell Solar Materials. N,N-dimethylformamide (DMF, 99.8%) and chlorobenzene (CB, 99.5%) were purchased from Acros Organics B.V.B.A. The precursor solution of perovskite was prepared by adding 1.8M PbI_2, 3.96M FAI, in 1 ml of DMF and stirring for 2 hours. 60 μL of the solution was spin coated on ITO substrates at 3000 rpm for 45. At the 5th second, 150 μL CB were deposited on

the spinning substrate. The resulting films were immediately annealed at 140 °C for 10 min in a glovebox.

Materials Characterization. The UV-Vis transmittance spectra of the perovskite films were recorded with a Carry 60 Agilent UV-Vis spectrometer. Steady-state photoluminescence spectra were recorded on a Horiba Fluoromax+ spectrofluorometer, equipped with a 150-W Xe lamp as the excitation source and a Hamamatsu R13456 PMT (185-980 nm nominal) as the emission detector. The crystal structure of the perovskite films was analyzed by the recorded XRD patterns, which were obtained with a SmartLab Rigaku diffractometer. The XPS data were collected with a PHOIBOS 100 (SPECS) hemispherical analyzer using Mg Ka X-ray source with photon energy 1253.64 eV. Voigt functions were used for the fitting analysis after standard Shirley background subtraction. The TEM measurements were performed using the FEI CM20 equipment at a beam voltage of 200 kV. The SEM images were acquired using the Jeol JSM 7401F equipment at a beam voltage of 2 kV.

Device Fabrication and Optoelectronic Measurements. ITO (40 nm) was sputtered on m-glass are room temperature using a high purity ITO target (purity 99.99%). Then, the substrates were cleaned by sonication washing with detergent (Helmanex III 2% in deionized water), deionized water, acetone, and isopropanol for 15 min. Before perovskite deposition, the cleaned ITO substrates were treated with ultraviolet ozone for 30 min and then transferred immediately into a N_2 glove box. The total thickness of the FA_2PbI_4 film was about 600 nm, while the thickness of the RF sputtered SiO_2 layer was about 10 nm. Finally, to obtain the complete devices, 40 nm of patterned Ag electrodes through a shadow mask were sputtered. The electrical measurements were performed at room temperature with the Keithley 4200

semiconductor parameter analyzer (4200-SCS) at the SUSS MicroTec probe station. The triggering of the RGB LED array was conducted using the HP 8116A Pulse Generator.

SUPPORTING INFORMATION REFERENCES

SI.1. Bricalli, A.; Ambrosi, E.; Laudato, M.; Maestro, M.; Rodriguez, R.; Ielmini, D. Resistive switching device technology based on silicon oxide for improved ON–OFF ratio—Part I: Memory devices. *IEEE Trans. Electron Devices* **2018**, 65, 1, 115 – 121.

SI.2. Yang, H.; Zhang, Y.; Hu, F.; Li, Z.; Wu, D.; Chen, X. Comprehensively Modulated Sub-Attojoule Operated Optoelectronic Synapses for Image Encryption and Inpainting, *ACS Appl. Mater. Interfaces* **2024**, 16, 42, 57804–57815.

SI.3. Kim, J.; Song, J.; Kwak, H.; Choi, S.-W.; Noh, K.; Moon, S.; Hwang, H.; Hwang, I.; Jeong, H.; Choi, S.-Y.; Kim, S.; Kim, J.K. Attojoule Hexagonal Boron Nitride-Based Memristor for High-Performance Neuromorphic Computing, *Small* **2024**, 2403737.
